# Ligand Versatility and Resistance Mechanism of Monotherapy-Grade HIV‑1 Protease Inhibitor GRL-142 Binding the Multidrug Resistant Variant p51: Insights from 1 μs MD Simulations

**DOI:** 10.1021/acs.jcim.5c02652

**Published:** 2026-02-27

**Authors:** Alejandro Arias, Chiara Cappelli, Albeiro Restrepo, Jorge Alí-Torres, Sara Gómez

**Affiliations:** † Instituto de Química, Universidad de Antioquia UdeA, Calle 70 No. 52-21, Medellín 050001 Colombia; ‡ Scuola Normale Superiore, Classe di Scienze, Piazza dei Cavalieri 7, Pisa 56126, Italy; § Departamento de Química, 28021 Universidad Nacional de Colombia, Sede Bogotá, Bogotá 110111, Colombia

## Abstract

The emergence of HIV-1 highly resistant strains and the prevalence of HIV-associated neurocognitive disorders (HAND), are two of the biggest challenges posed to combination antiretroviral therapy (cART), despite promising advances in treatment. To address these issues, the protease inhibitor GRL-142 (G), an extremely potent and central nervous system (CNS)-penetrating antiretroviral, has recently been experimentally proposed as monotherapy and enhanced cART efficacy against HAND. Using all-atom molecular dynamics (MD) simulations of up to 1 μs, this study elucidates the energetics, dynamics, and bonding interactions that govern the inhibitory mechanism of G against the highly resistant HIV-1 protease, p51, for which it exhibited the lowest experimental potency. Our MD trajectories allow us to capture the complex structural and dynamical interplay between this state-of-the-art inhibitor and p51. The protein mechanism of resistance involves retention or even improvement of structural stability at key active regions, expansion of its active site cavity, and disruption of the HB network with the inhibitor, compared to the wild-type (Wt) complex. As a consequence, the inhibitory backbone binding mechanism of G is lost at the P2′ functional group moiety. Yet, G engages in direct drug–protein interactions that compensate for the loss of the crystallographic flap-water and undergoes a binding mode transition, preserving important interactions to the inhibitory mechanism. Conserved fluorine-mediated interactions help stabilize both Wt–G and p51–G complexes. The calculated MMPBSA binding energy of Wt–G during the entire trajectory is in close agreement with the experimental value (Δ*G*_MMPBSA_ = −16.1 kcal mol^–1^ vs Δ*G*_exp_ = −14.9 kcal mol^–1^). For the Mut–G system, there is slightly less affinity with Δ*G*_MMPBSA_ = −15.5 kcal mol^–1^. The novel binding mode of G in p51–G has a higher affinity of (Δ*G* = −18.4 kcal mol^–1^), which highlights its relevance from a structure-based drug design perspective and the structural versatility of inhibitor G. Despite this energetic favorability, the detachment of P2′ from its canonical subsite, disrupts key pharmacophoric interactions and the bioactive conformation required for inhibition, indicating that optimization of P2′ is needed to preserve the backbone binding mechanism against highly resistant strains.

## Graphical abstract

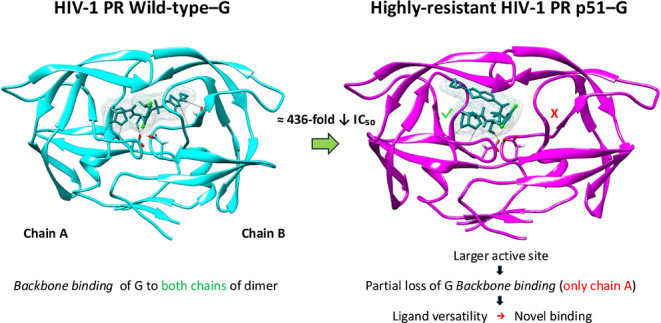

## 1 Introduction

Combination antiretroviral therapy (cART) constitutes the greatest breakthrough in the fight against human immunodeficiency virus (HIV) and the acquired immunodeficiency syndrome (AIDS) epidemic, and it remains the standard treatment to this day. Its success has led to a reduction in morbidity, mortality, and a significant improvement in quality of life in infected patients, providing them with life expectancies comparable to those of uninfected individuals.^1–4^ Despite these advancements, HIV/AIDS continues to be a major global health concern, with an estimated 40.8 million people living with the virus, 1.3 million new infections, and, tragically, 630000 deaths related to AIDS during 2024.^5^ In addition, several limitations persist in the currently available cART regimens, which become particularly significant given the absence of a definitive cure (in the short term) for HIV and the need for lifelong adherence.^6^ The foremost obstacle in cART therapy is arguably the high rate of viral mutation, a hallmark of HIV, driven by its error-prone reverse transcription process, which leads to the emergence of highly resistant strains. The prevalence of HIV-associated neurocognitive disorders (HAND) poses another critical challenge to therapy,^7–9^ probably related to poor central nervous system (CNS) penetration of antiretroviral agents that turns the brain into a reservoir of virus.^10,11^ Other challenges include toxicity and secondary effects.^12,13^

cART regimens consist of a cocktail of antiretrovirals that block key stages of viral replication, which is mediated by the activity of the three main HIV enzymes: reverse transcriptase (RT), integrase (IN), and protease (PR). Among them, PR is a fundamental pharmacological target in cART therapy because it is responsible for the processing of the Gag and Gag-pol polyprotein precursors that permits the assembly of mature, infectious viral particles. A model PR structure is shown in Figure 1a. It consists of a homodimer with 99-residue per monomer (chains A and B) and contains six main structural regions, including highly dynamic flaps that regulate substrate and drug entry to an aspartyl-like active site, among other structurally mobile regions.

**Figure 1:**
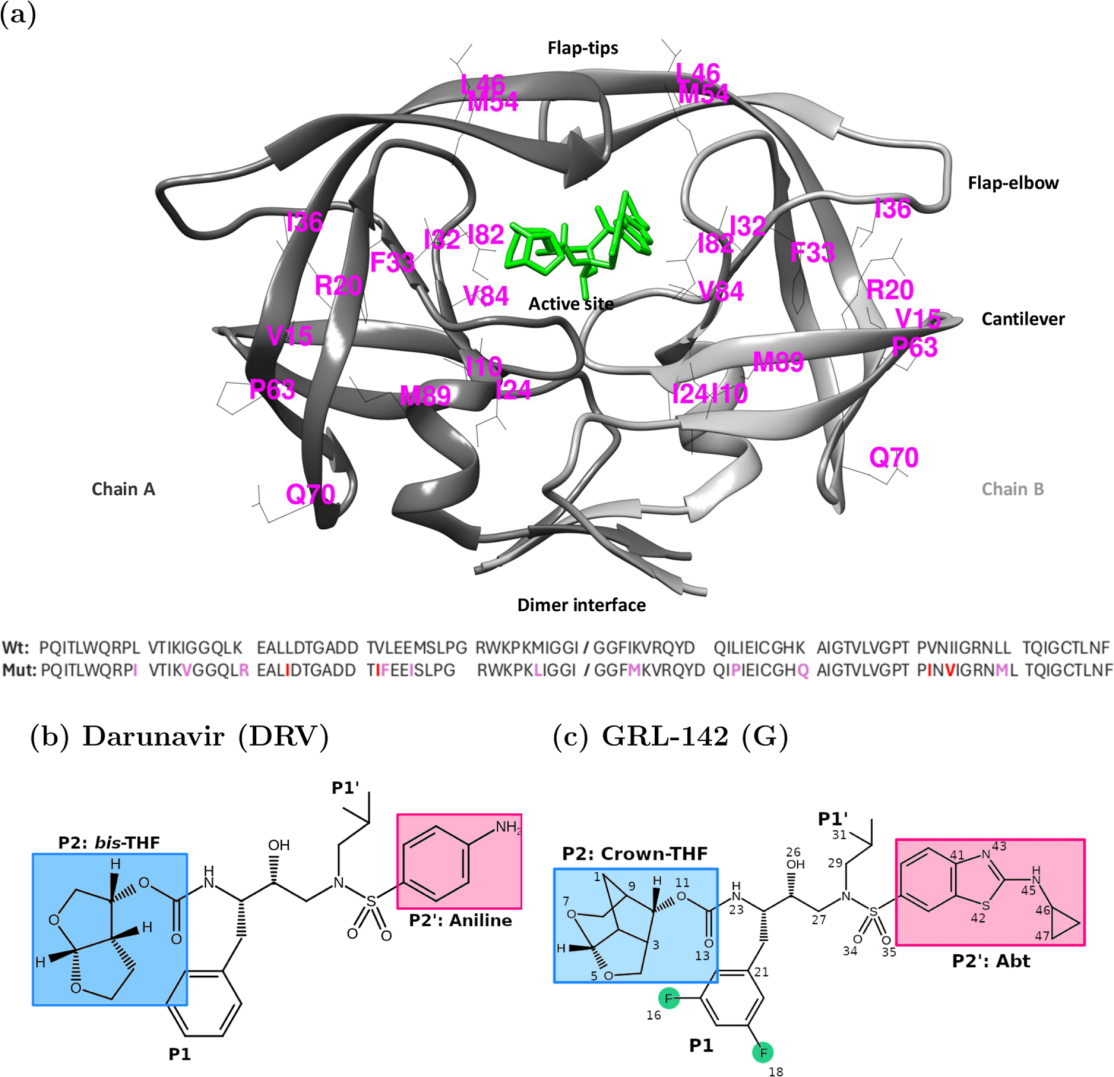
(a) Molecular structure of HIV-1 PR p51 (Mut). Mutated residues in which it differs from Wild-type HIV-1 PR (Wt) can be seen in the structure and in the sequences below. The six main structural regions are indicated. GRL-142 (G) is placed in the active site to indicate the native binding mode of the ligand. (b) Darunavir (DRV) and (c) G structures.

To block PR activity, a series of protease inhibitors (PIs) has been developed. They are among the cART antiretrovirals with the highest genetic barriers, a property that limits or prevents the viral evolution of resistance-associated mutations, which in turn weaken drug–target interactions and render treatment ineffective. Darunavir (DRV) is the latest and most potent FDA-approved PI in clinical use, and the only one that continues to be recommended as an option for first-line therapy.^14–18^ DRV also proved to be effective against highly multidrug-resistant HIV-1 strains.^19^ Its high potency has been attributed to its dual mechanism of action, that involves inhibition of both the active HIV PR homodimer and the dimerization process itself.^20^

The rationale behind the DRV design is to optimize the specific interactions of earlier PIs, which already achieve molecular recognition by mimicking the natural substrate within the active site.^21^ More specifically, the conformations of the backbone atoms of the protease active site do not change significantly between Wild-type proteases (Wt) and mutant proteases (Mut). Hence, any PI capable of establishing extensive HBs with the active site backbone of Wt will have the best chances to maintain efficacy against Mut, thereby impairing the emergence of resistance. This is one of the most successful and well-established structure-based drug designs and has been described as the backbone binding strategy.^22–25^ By virtue of this design, DRV, shown in Figure 1b, contains the bis-tetrahydrofuran, bis-THF, moiety (backbone binder P2 moiety) to interact with backbone atoms of residues D29 and D30 in chain A. Additionally, a 4-aminobenzenesulfonamide (backbone binder P2′ moiety) addresses residue D30 in chain B. These structural functionalities in DRV are paramount for enhancing not only the formation of strong HBs but also van der Waals interactions with Wt active site residues, ensuring antiviral efficacy.^26,27^

Notwithstanding the elaborate structural optimization that resulted in the high potency of DRV, it cannot be used as monotherapy. DRV must be administered with Ritonavir, a booster PI, to ensure optimal systemic concentrations.^28^ Moreover, DRV is used in combination with other antiretrovirals in the context of cART to achieve an efficient regimen.^29^ The most serious challenges posed to DRV treatment are the emergence of highly DRV-resistant strains, identified both in vitro and in vivo.^30,31^ Furthermore, DRV concentrations in the CNS are suboptimal due to poor penetration, making it ineffective in addressing latent forms of the virus in the brain, which may be related to the prevalence of HAND.^9^

In response to the need for improved PIs, the group of Gosh et al. further optimized structural features of DRV under the backbone binding strategy, arriving at GRL-142 (G), shown in Figure 1c, a next-generation PI candidate that incorporates the novel crown-like tetrahydropyrano-tetrahydrofuran (crown-THF) as the P2 moiety, replacing bis-THF of DRV, and the aminocyclopropylbenzo-thiazole (Abt) sulfonamide at P2′, replacing aniline in DRV. Additionally, and of particular significance, the P1 benzyl fragment of DRV was replaced by a bis-Fluoro-benzyl (bis-FBz) group to potentiate CNS penetration and address HAND prevalence.^32^ The implementation of fluorine in medicinal chemistry has seen enormous growth in the last decades due to its beneficial properties, including lipophilicity, which enhances membrane permeability, improved metabolic stability, and establishment of novel attractive halogen interactions that may increase the potency of pharmaceuticals.^33–38^

G displayed an exceptional inhibitory activity with a *K*_*i*_ value of 14 pM and reported IC50 values of 0.0094 nM,^39^ 0.016 nM,^40^ and 0.019 nM,^25^ against Wt strains, representing 542-fold, 231-fold, and 168-fold enhancements in potency, respectively, compared to the corresponding values of DRV. When tested against the highly-DRV-resistant strains p20, p30, and p51, G exhibited a markedly improved activity compared to a wide range of PIs, including DRV, making it the best PI against these highly HIV-1 resistant strains that have been reported.^41^ Strikingly, G exhibits comparable potency against p30 relative to Wt, and an even higher potency against p20. This remarkable inhibitory performance led the team behind G design to propose it as the first PI to be developed as an HIV-1 monotherapy.^25,32^ Among the resistant variants, only p51 showed a decrease in viral potency of G compared to its potency against Wt, with reported 436-fold,^39^ 175-fold,^40^ and 63-fold decreases.^40^ Although there is no rigorous consensus on the differential IC50 values of G across studies, it is clear that G outperforms DRV against all tested strains, with its only limitation lying in a reduced activity against p51.

Accordingly, this study aims to determine the energetic, dynamical, and structural factors underlying the binding of G to Wt and to the highly DRV-resistant p51, as these are central to understanding and explaining the differences in their reported antiviral potencies. To this aim, we have performed up to 1 μs all-atom molecular dynamics (MD) simulations of Wt–G and Mut–G complexes to best approach the motion of structural protein domains that influence drug binding. We pinpoint the specific interactions that drive the strong binding of G and determine the role of the fluorine-mediated interactions. This information is valuable for the elucidation of the molecular mechanisms in p51 that account for its highly resistant profile. Additionally, a comprehensive understanding of the binding interactions of G with its target is crucial for guiding its further optimization as a monotherapy and for the general purpose of designing improved PIs for cART.

## 2 Methods

### 2.1 System Selection

There are two species of HIV, namely, HIV-1 and HIV-2.^42^ HIV-1 group M (main) is the most infectious and responsible for the ongoing HIV/AIDS epidemic, having caused more than 90% of all infections worldwide.^43^ HIV-1 group M divides further into a range of subtypes, including A-D, F–H, J, K, and a variety of circulating recombinant forms (CRFs) and unique recombinant forms (URF).^44^ Subtype B is the most studied, so it offers the largest amount of reliable data to validate against. In addition, subtype B infection has been the main cause of epidemics in America, specifically in Latin America. Accordingly, we have selected the wild-type protease from HIV-1 group M subtype B as our general wild-type protease model (Wt). The mutant p51 is a highly resistant strain to clinically used PIs and reverse transcriptase inhibitors (NRTIs). In fact, p51 has been established as the most multi-PI-NRTI-resistant and DRV-resistant HIV-1 variant.^25,30^ Furthermore, Ligand G exhibits the same IC50 value against Wt PR, and other highly resistant strains like p20 and p30.^32^ However, it shows up to 436-fold decrease in activity when faced with p51, making it a suitable model to study resistance to G. Thus, we selected p51 as our model for a highly resistant protease (Mut).

### 2.2 System Preparation

The three-dimensional structures for the Wt–G and Mut–G complexes were obtained from the Protein Data Bank (PDB) entries PBD: 5TYS and PDB:6MKL, respectively.^32^ The HIV-1 PR active site residues D25 A and D25 B are crucial in the catalytic activity of the enzyme and have been reported^45^ to exist in a monoprotonated state at the delta oxygen OD2 of D25 in chain B (see Figure 1a). Accordingly, the CHARMM-GUI PDB reader and manipulator module was used to set this protonation state, as well as to add Wt and Mut missing hydrogen atoms (pH = 7.4) and define terminal groups.^46–48^ The ff14SB^49^ force field was used to generate the protein topologies. All MD calculations were performed with the GROMACS 2020.4 suite.^50–52^

The geometry of the G ligand was obtained from the 5TYS complex. Missing hydrogen atoms were added for pH = 7.4 with the Open Babel^53,54^ extension within Avogadro,^55^ and then the molecule was optimized at the ωB97XD/aug-cc-pVDZ^56,57^ level using Gaussian16.^58^ Once the protein and ligand were prepared as mentioned before, we performed molecular docking calculations in Chimera + vina^59,60^ and SwissDock^61,62^ to select the starting geometry for the drug···protein complex during the MD runs. Conditions for molecular docking calculations can be consulted in Section 1 (“Docking details”) of the Electronic Supporting Information (ESI). The ligand GROMACS-compatible topology was generated using the Antechamber python parser interface (acpype)^63–65^ with the general amber force field 2 (GAFF2) parameters.^66^ Hirshfield charges (CM5) were used in the ligand topologies.^67–70^ Periodic boundary conditions were accounted for in a rhombic dodecahedral periodic box, with a minimum solvent-box distance of 1.0 nm.

The complexes were solvated with the TIP3P water model^71^ in their respective boxes. Na^+^ and Cl^–^ ions were added to neutralize the system and ensure physiological salt concentrations (0.15 M NaCl). At this stage, 10496 water molecules, 32 Na^+^, and 37 Cl^–^ atoms were added to Wt-G; as for the Mut-G system, the corresponding numbers were 11697, 35, and 38, respectively. Finally, the energy of both systems was minimized using the steepest descent algorithm.

### 2.3 Molecular Dynamics Simulations

First, both Wt-G and Mut-G systems were equilibrated in the *NVT* ensemble for 0.5 ns. The v-rescale algorithm^72^ was used to couple the temperature to an external heat bath, allowing the system to reach thermal equilibration at 310 K. Then, a 2 ns *NPT* equilibration was conducted, coupling the system to a Berendsen barostat.^73^ During this equilibration, position restraints were gradually released in four successive intervals of 50 ps each (force constants of 1000, 100, 10 kJ mol^–1^ nm^–2^, and finally, no restraints) to allow a progressive relaxation of the systems. Finally, two MD replicas of 500 ns were conducted for the Wt–G system, and three MD replicas of 1 μs were run for the Mut–G system. Each system evolved in the *NPT* ensemble at 310 K and 1 bar, using the Parrinello–Rahman barostat,^74^ with a print interval of 20.0 ps for a total of 25000 frames. To the best of our knowledge, the only MD study of G has implemented up to 100 ns.^75^ The reason to perform a third replica in Mut–G was to improve the statistical reliability of all the descriptors, particularly the binding energy calculations, given that no experimental value is reported for such system.

Despite HIV-1 PR···PI complexes being regarded as relatively small systems, it has been established in the literature that the flap regions are mobile on the microsecond scale, and the flap-tips present mobility on the subnanosecond scale.^76,77^ Shabanpour et al.^78^ reported that the intricate dynamics of the complexes require at least 200 ns length MD simulations to capture in detail domain motions. Other studies of the apo HIV PR previously reported that flaps remain in a closed conformation during ≈400 ns, and then open beyond this time.^79^

For both *NVT* and *NPT* equilibrations and MD runs, the following parameters for noncovalent interactions were applied: The Verlet scheme was used for neighbor searching, employing the grid method to determine neighbor lists. The particle mesh Ewald (PME) method^80^ was used to treat long-range electrostatic interactions, using a 1.2 nm cutoff. The same cutoff was applied for short-range van der Waals interactions.

All structural analyses, including the root-mean-square deviation (RMSD), the root-mean-square fluctuation (RMSF), radius of gyration, number of HBs, etc., were performed on subtrajectories containing 5000 (or 10000) frames from the entire MD runs. To determine the number of HBs, we used the same geometrical criteria employed in our previous works studying virus···cell receptor interactions.^81,82^ The VMD 1.9.3 software^83^ was used to calculate the occupancies of the fluorine-mediated interactions, as well as the distance criteria to define the conformations of the protease flap-tips. The CASTpFold engine was used to compute the active site volume. CHIMERA 1.17.3 ^59^ and VMD were used for visualization and graphical representations.

### 2.4 Binding Energy Estimation via Molecular Mechanics–Poisson–Boltzmann Surface Area (MMPBSA)

The binding energies of the Wt-G and Mut-G complexes were calculated within the MMPBSA methodology. For such a purpose, we used the gmx_MMPBSA software,^84^ which enables the postprocessing of GROMACS MD trajectories to be implemented in the AMBER MMPBSA.py program.^85^ For each system replica, a total of 200 frames were analyzed by using the 5000 frames subnanotrajectory, and further dividing it into 25 frame intervals. The values of the external (solvent) and internal (solute) dielectric constants were set to 80.0 and 1.0, respectively. Ionic strength of 0.15 M and a 310 K temperature were applied to best mimic physiological conditions. Additionally, the entropy corrections were computed using the Normal mode analysis of the vibrational frequencies (NMODE), with a maximum number of minimization cycles per snapshot of 10000, a convergence tolerance for minimized energy gradient of 0.01 kcal mol^–1^ Å^–1^, and the same ionic strength used for the energy calculation. To aim for a more rigorous representation of nonbonded energy contributions, a decomposition scheme with 1–4 electrostatic interactions added to the electrostatic potential, and 1–4 van der Waals interactions added to the VDW potential was selected.

## 3 Results and Discussion

The following sections present the structural, dynamic, and energetic analyses of GRL-142 (G) binding to Wt and highly drug-resistant p51 mutant HIV-1 proteases. Molecular docking results can be consulted in Section S1 in the ESI. Herein, we first describe the conformational dynamics analyses through RMSD, RMSF, and active site volume calculations. Subsequently, we characterize the bonding interactions governing inhibition, including hydrogen bonding networks, flap-water mediated interactions, and fluorine-mediated contacts. Finally, we report MMPBSA binding free energies to provide thermodynamic insights into the observed binding modes and resistance mechanisms.

### 3.1 Structural Analysis

#### 3.1.1 Conformational Analysis of Wt and Mut Proteases

The RMSD of the backbone atoms in Wt and Mut proteases was calculated to evaluate their global structural stability within the drug–protein complexes during the MD simulations. As can be seen in Figures 2a,b and S1a in ESI, both proteins reach stability early in the simulation, regardless of the replica. In the 0–500 ns interval, the mean RMSD and standard deviation values were, for Wt, 0.1011 ± 0.0108 nm in Rep1, 0.1054 ± 0.0105 nm in Rep2, and for Mut 0.1145 ± 0.0129 nm in Rep1, 0.1128 ± 0.0108 nm in Rep2 and 0.1154 ± 0.0146 nm in Rep3. The small mean RMSD increase in Mut, observed in Rep1 and Rep2, with respect to Wt, may be ascribed to its behavior during the 450–500 ns interval of simulation, where a subtle rise can be observed in both replicas. Moreover, the radius of gyration (*R*_g_) (Figure S3 in the ESI) indicates that the overall protein structures of Wt and Mut complexes remained compact during the entire simulation. After an MD extension up to 1 μs for Mut–G systems (see Figure S1a in ESI), it can be observed that the RMSD increase in Mut–G during the 450–500 ns interval reaches a small peak at ≈500 ns, in replicas 1 and 2, after which the initial stable mean value is regained. The radius of gyration is also stable during the extension, as can be seen in Figure S1e in ESI.

**Figure 2:**
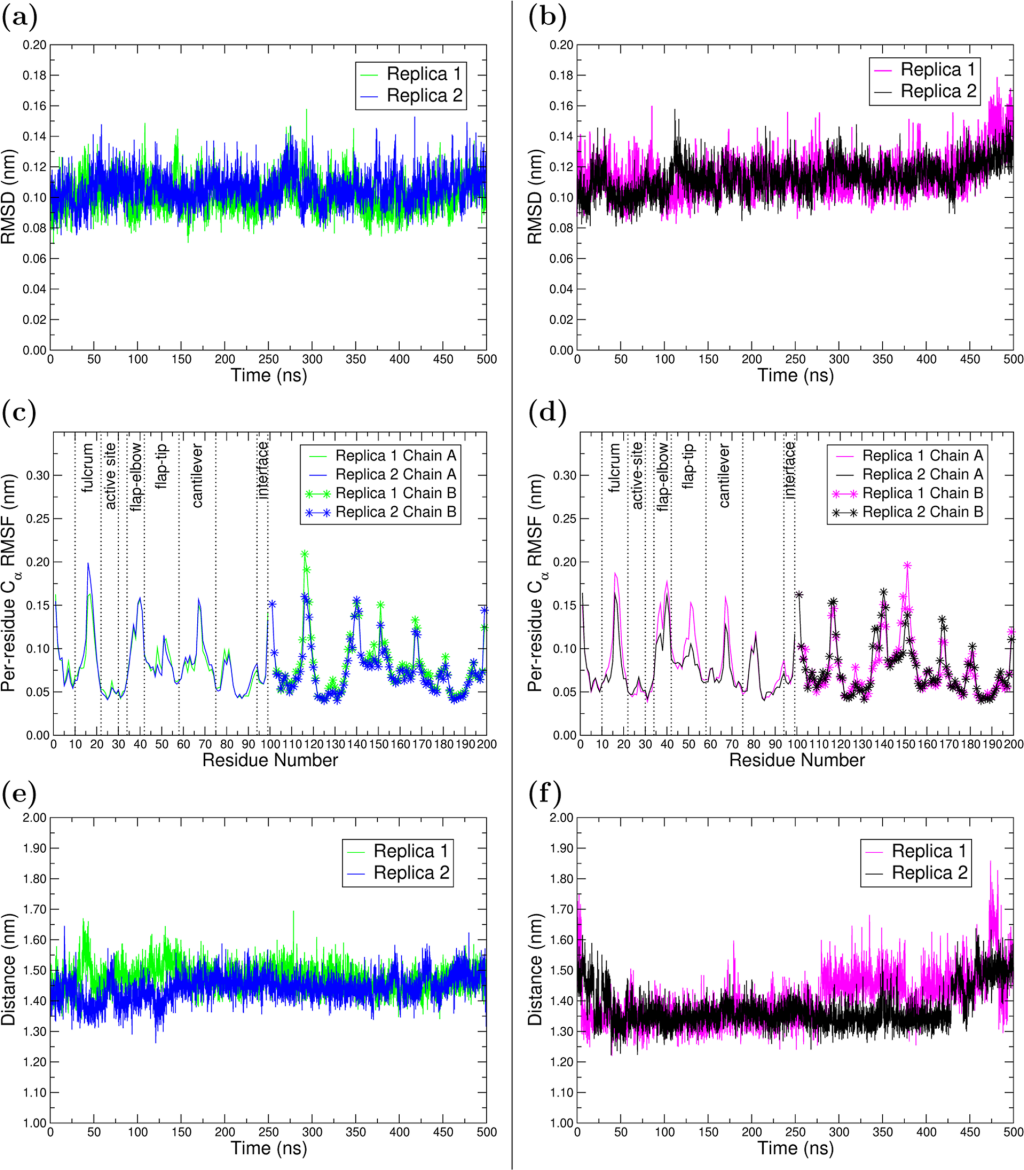
Conformational analysis over the MD trajectory for HIV-1 Wt (left) and Mut (right). (a,b) Time evolution of the RMSD of PR backbone atoms. (c,d) RMSF of C_α_ in PR. The symmetrical, main structural regions are only indicated for chain A (residues 1–99). (e,f) Time evolution of the distance criterion D2 (D25 A −I50 A inter-residue distance) to monitor active cavity volume. Criteria D1, D3, and a schematic representation depicting all three criteria in PR can be consulted in Figure S5a–d, in the ESI, respectively.

To approximate the structural and dynamical factors behind Mut RMSD behavior during the 450–500 ns interval of simulation, we superimposed protein structure at 450 ns, where stability had been reached, with structure at 500 ns, where RMSD had shifted upward (Figure S4 in the ESI). By comparing both structures, it can be observed that during this interval, a significant displacement of the flap-elbow, fulcrum, cantilever, and flap-tip regions is occurring (see Figure 1a). Furthermore, a small displacement of the active site can also be detected.

To gain further insights into the structural dynamics of the Wt and Mut proteases, RMSF plots highlighting the main structural region, as shown in Figure 2c,d, allow distinguishing between highly fluctuating and rigid regions. These structural elements are equivalent across chains A and B of the dimer, and comprise the fulcrum (11A–22A and 11B—22B), active site (23A–30A and 23B–30B), flap-elbow (35A–42A and 35B–42B), flap-tip (43A–58A, 43B–58B), cantilever (59A–75A, 59B–75B) and interface (95A–99A and 95B–99B).^78^ In both Wt and Mut, the fulcrum, flap-elbow, cantilever, interface, and flap-tips are characterized as highly flexible. Notably, the flap-tip in chain B of Mut–G (in both replicas) is showing a higher fluctuation, compared to the flap-tip in Chain A, and also compared to both flap-tips in Wt–G. This result indicates that an asymmetrical motion of the flap tips is occurring in Mut–G, with flap-tip in Chain B being the most flexible. In contrast, the active-site regions show significantly lower flexibility, which is consistent with the expected structural stabilization arising from interactions with the ligand (moieties P1, P2, P1′, P2′).

Along with the active site, the flap-tips are crucial structural regions for enzymatic activity, given their complex dynamics regulate the entry of the natural substrates and, consequently, of the PIs to the active site.^86–90^ It has been experimentally and computationally demonstrated that in the unbound state, HIV-1 PR exists in an equilibrium ensemble of four different flap conformations: open, semiopen, closed, and curled open, with semiopen being the predominant conformation.^91,92^ Various interatomic C_α_ distances throughout the active site have been proposed as reasonable metrics to distinguish between flap conformations during MD runs, including D1 (I50A–I50B), D2 (D25A–I50A), and D3 (D25B–I50B) distances. Accordingly, to analyze the important motion of flap-tips, Figures 2e,f and S5 in the ESI show D1, D2, and D3 criteria for both systems during the MD run. For the Wt–G case, each of the three distance criteria remains constant throughout the first 500 ns MD, indicating no significant changes in the flap-tips conformations, nor in the active site cavity volume take place. By contrast, the corresponding plots for Mut–G exhibit fluctuations in several regions, specifically D2 and D3 criteria. In terms of flap-tip conformations, approximately constant D1 values in Wt–G and Mut–G (Wt:0.7 nm, Mut:0.6 nm, Figure S5a,b) indicate that both systems retain the closed conformation along the simulation time.^89,91^ A shorter distance of flap-tips in the Mut case has been previously observed in the structurally related complexes with DRV.^78^ Our D1 values are also consistent with those of the corresponding crystal structures, 0.6103 and 0.5767 nm for Wt-G and Mut-G, respectively. As revealed by the 1 μs Mut–G MD extension, D1, shown in Figure S1f of the ESI, remains approximately constant throughout the entire simulations. Thus, the flap-tips closed conformation is stable in Mut–G, and should not be crucial in its resistance mechanism.

The D2 and D3 criteria, which correspond to the distance between the active site aspartates and the flap-tips, remain stable in Wt-G, with mean values of 14.7 ± 0.4 Å for D2 and 14.5 ± 0.5 Å for D3 in Rep1 and Rep2, respectively. However, a comparatively larger variation is observed in Mut-G, especially during the 450–500 ns interval. Given that the active site region is rigid (see Figure 2c,d), this increase in D2 and D3 implies an upward motion of the flexible flap-tips, and thus an enlargement of the active site cavity in Mut–G. Expansion of the active site cavity in HIV-1 PR is a known form of drug resistance.^93^ The MD extensions to 1 μs show that D2 reaches a peak at around 500 ns in both replicas, and then slightly decreases during the next 400 ns, to increase again from 900 ns up to 1 μs (Figure S1g of ESI). Therefore, a fluctuating active site expansion is taking place during the simulation, in the D25A–I50A direction. Regarding D3, after slightly increasing toward 500 ns and experiencing fluctuations around this value, it stabilizes beyond 600 ns up to 1 μs (Figure S1h of ESI). These expansions may allow the ligand to adopt new conformations and explore additional regions within PR.

Subsequent calculations with the CASTpFold engine^94^ allowed verification of the volumen expansion inference. For the Wt–G systems, the final volumes at 500 ns were 498.7 and 475.5 Å^3^, and the corresponding values in Mut–G were 695.7 and 553.9 Å^3^ (Figure S6a,b in ESI). This represents an expansion of the Mut-G active site as large as 220.2 Å^3^, with respect to Wt-G. Volumes for Mut–G Rep1 were then computed at 800 ns and at 1 μs (Figure S6c,d). It can be seen that the volume increases to a value of 885.4 Å^3^ at 800 ns, and then decreases to 644.3 Å^3^ at 1 μs. This behavior is consistent with the time evolution of D2 and D3 beyond 500 ns.

#### 3.1.2 Conformational Analysis of Ligand G: RMSD, RMSF, and Identification of Binding Modes

The RMSD for G was calculated to assess ligand structural stabilization (Table S1 and S7a in the ESI and Figure 3a,b). In both Wt-G replicas, G reaches relative stability from 150 ns onward, with mean RMSD values of 0.1307 ± 0.0210 nm and 0.1505 ± 0.0201 nm, for Rep1 and Rep2, respectively. The corresponding mean RMSD and standard deviation values for Mut-G replicas were larger, namely, 0.2282 ± 0.1004 nm and 0.2070 ± 0.0617 nm in the 0–500 ns interval. The value for Rep3 was 0.2850 ± 0.0715 nm.

**Figure 3:**
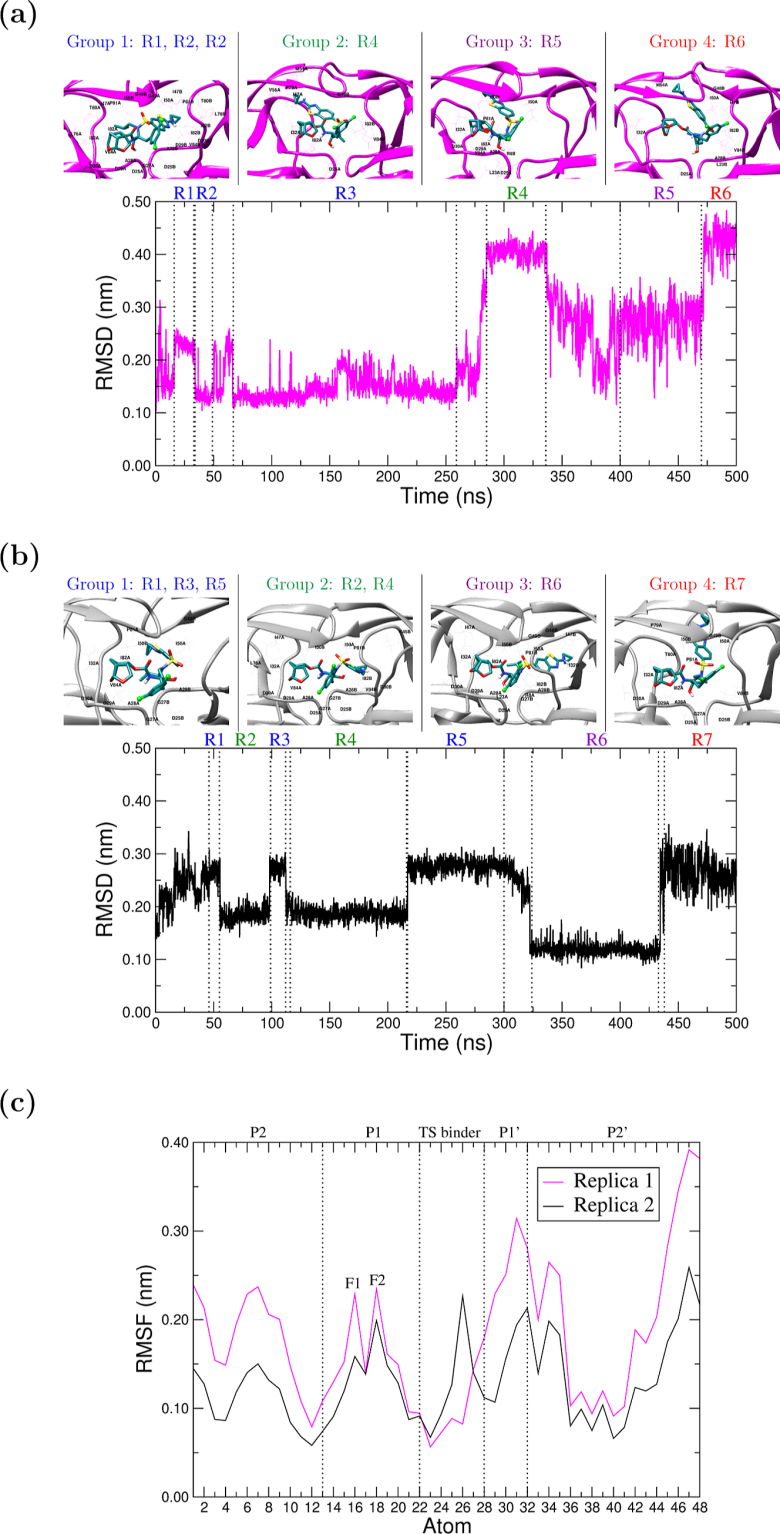
Conformational analysis of G in MUT-G. (a) Ligand RMSD plot for Rep1. (b) Ligand RMSD for Rep2. (c) Ligand RMSF for both replicas. On top of each RMSD plot (a,b), a representative structure of the complex is shown for each group. The regions comprised by each group can be identified by their color. Amino-acids within 2.0 Å of G are indicated.

In the three replicas, the ligand explores new conformational regions that deviate from the reported bioactive conformation, which could provide clues to understand resistance in Mut. In the following, we will focus on the analysis of Replicas 1 and 2, which consistently end in a similar binding mode (see Figure 4). The final conformation of G in Rep3 can be consulted in Figure S12 in the ESI.

**Figure 4:**
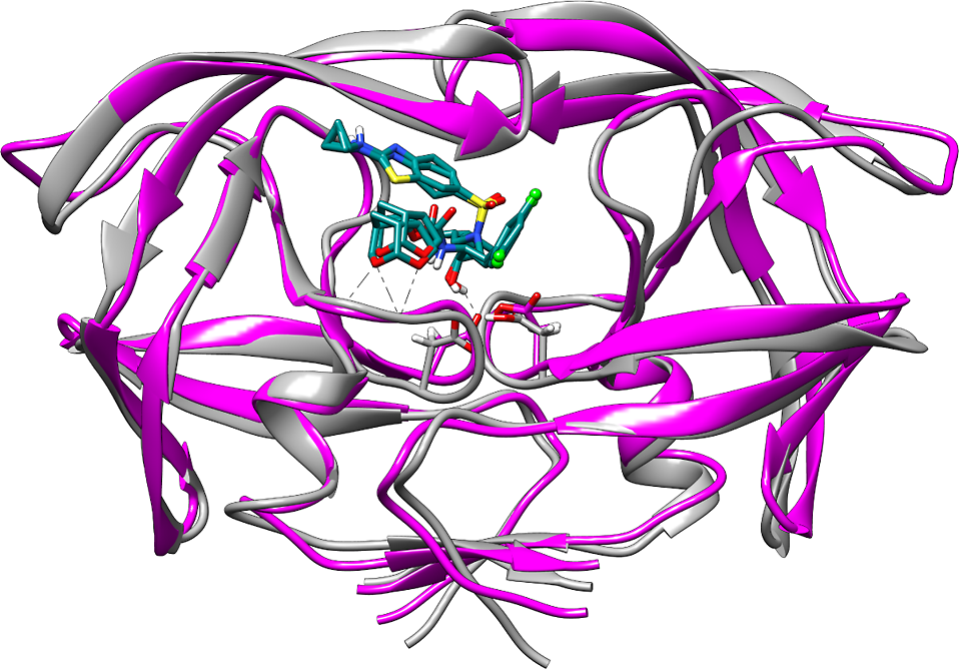
Superposition of the final binding modes of G at 1 μs in Rep1 and Rep2 of Mut–G system, showing an alternative conformation of G which is consistent beyond 600 ns.

The RMSD plot of the ligand in Mut-G reveals sharp jumps between various RMSD plateau regions, where relatively stable poses are maintained. Given the structural stability of Mut during the MD simulation, as discussed in the previous section, these regions provide information on the binding modes of G within Mut. However, careful consideration is required, since very small changes in RMSD may give rise to structurally different ligand poses, or similar poses in different protein pockets/active site conformations. Moreover, it needs to be considered that these configurations reflect relatively stable binding modes in a dynamic pocket, because the Mut structure, though little compared to the ligand, is also experiencing structural changes in time, especially during the 450–500 ns interval of the simulation. On this basis, after closely examining both the orientations of the ligand and the protein in the previous regions, we gathered them into groups of similar binding and ultimately into two main binding modes, referred to as native-like and P2′-withdrawal as can be observed in Figure 3a,b and Table 1.

**Table 1:** Classification of Ligand RMSD Plateau Regions in Groups of Similar Binding for 0–500 ns Interval^b^

replica	binding	group	region	interval	Δ*H*	*T*Δ*S*	BE for	BE for
	mode			(ns)			region^a^	binding mode
1	native-like	1	1	16–33	–52.8	29.3	–23.5	–17.2
			2	34–49	–47.2	33.2	–14.0	
			3	67–259	–50.3	33.2	–16.9	
	P2′-withdrawal	2	4	285–336	–48.5	32.3	–16.3	–12.6
		3	5	400–470	–42.9	32.7	–10.2	
		4	6	470–500	–41.5	29.5	–12.0	
2	native-like	2	2	55–99	–47.5	26.2	–21.3	–23.2
			4	116–216	–54.3	29.0	–25.3	
		3	6	324–433	–56.8	32.2	–24.6	
	P2′-withdrawal	1	1	46–55	–45.4	29.1	–16.3	–21.2
			3	99–112	–45.7	28.1	–17.5	
			5	217–300	–51.2	28.3	–22.9	
		4	7	438–500	–44.1	31.1	–12.9	

a For Region 8 (R8), beyond 600 ns in the 1 μs extension of the MD runs, Δ*G*_Mut_Rep1_R8_ = −17.2 and Δ*G*_Mut_Rep2_R8_ = −19.6 kcal/mol, Figure S1b in the ESI.

b Two major binding modes, referred as native-like and P2*′*-withdrawal, were found. Binding energies (BE) with their contributing terms (*ΔH* and–*T*Δ*S*) are reported for each region in kcal/mol. The weighted averages are based on time occupancies (see Tables S6 and S7 in the ESI) are reported for each binding mode.

To achieve this structural classification, we carefully analyzed each interval and its limits by superimposing structures every 10 ns throughout the entire trajectories. In the case of Rep1, intervals R1, R2, and R3 comprising the first 250 ns of simulation may be grouped (group 1) in a conformation akin to the native one. Afterward, a critical detachment of the P2′ backbone binder is observed, while the backbone binder P2 and fluorinated P1 moieties remain in their corresponding subsites. P2′ alternates between different flap-tip regions as the active-site volume increases near the end of the 500 ns simulation. Consequently, in group 2 (R4), P2′ orients toward P79 and mutant M54 in chain A. In group 3 (R5), P2′ orients toward P81 in chain A and I50 in chain B. Finally, in group 4 (R6), P2′ is near the tip of chain B near residue I47, and also points toward residues I50 and mutant M54 of chain A.

In the case of Rep2, seven structural intervals were identified, gathered into 4 groups. During the first 45 ns of simulation, the ligand is unstable. Then, noticeably early in the simulation, P2′ loses its native interactions with D30B to first enter group 1 (R1, R3, and R5), characterized by P2′ interactions with flap-tip residues P81 A, A28 B, G48 B, and mutated I82 A. Then, in Group 2 (R2 and R4), the ligand adopts a conformation akin to the native. Group 3 (R6) is structurally similar to group 2, with a change in the orientation of the G hydroxyl. Finally, in Group 4 (R7), although similar in RMSD to group 1, P2′ is closer to the flap-tips between residues G49 B, T80 A, and P81 A. Beyond 600 ns in our MD extensions, the ligand adopts a stable non-native conformation in which P2′ is oriented to the flaps, consistent in both replicas. This new region is referred to as R8 in Figure S1b in ESI. A fluctuation of the ligand RMSD can also be observed when simulated in pure water, as shown in Figures S13 and S14 in the ESI. This points to the ligand G intrinsic structural versatility that is exploited in the resistance mechanism of the protease, given its expanded active site volume.

To summarize the previous observations, the groups identified in each replica were classified into two predominant binding modes, differentiated by the relative position of P2′ in PR: (*i*) A binding mode exhibiting native-like configurations (Rep1: group 1, Rep2: groups 2 and 3) characterized by an orientation of the P1, P2, P1′ and P2′ moieties similar to the one observed in the crystal structures of Wt–G and Mut–G (PDB:5TYS and PDB:6MKL, respectively), i.e, evidencing the backbone binding design; (ii) a binding mode with alternative P2′-withdrawal configurations, characterized by a critical withdrawal of P2′ from its native pocket to interact preferentially with the flap-tips, while residues P2 and P1 retain the contact with their native subsites. This binding mode of the ligand appears early in the simulations and is maintained during significant fractions of the simulation time.

The RMSF plots of the ligand G in Mut–G and Wt–G are presented in Figure S7b in the ESI and in Figure 3c. The P1′ moiety is the most mobile region in Wt–G, which is in line with its behavior in the structurally related Wt–DRV complex.^78^ However, while P2′ moiety is almost fixed in Wt–G complex, it surpasses P1′ in the Mut–G and becomes the most flexible region of the ligand.

It must be stressed that G was designed to maintain the binding mode named here as native-like both within Wt and Mut. The developers of G and similar PIs have referred to them as “molecular crabs”, which ultimately grab each HIV-1 PR monomer via their P2 and P2′ moieties. Not only experimental X-ray structures, but MD studies, including our own simulations, have shown that this native-like binding mode is stable in complexes of DRV, G’s template, with both Wt and Mut (see Figure 1). Most importantly, G was designed to enhance this backbone binding mechanism, and has been experimentally shown^25^ to have a 150-fold superiority over DRV to address highly-DRV resistant strains. Yet, early in our MD simulations, G is displaying binding modes different from the native-like (backbone binding one), which persist in significant fractions of the MD simulation. These interactions involve the flaps, which must be stabilized via water-mediated HB networks with the PI to ensure effective inhibition. There is experimental evidence that highlights the prominence of novel binding interactions of PIs in structure-based drug design. Zhang et al.^95^ have shown atypical binding modes of DRV to mutant p51-D25N, in binding modes that involve the flap-tips region, providing opportunities for the design of inhibitors targeting open PR conformations.

All the previous information suggests that (1) from the point of view of the backbone binding inhibitory mechanism, the P2′ moiety in G partially disrupts this mechanism, as it repeatedly detaches from its subsite. (2) The novel binding modes of G in Mut highlight its structural versatility and should not be overlooked, deserving a detailed analysis in the context of drug optimization, as they might play a role in G’s inhibitory activity. Previous studies of structurally related inhibitors of G have also pointed to structural plasticity.^96^

To delve deeper into these novel binding modes and how they relate to the backbone binding mechanism expected in G vs the resistance mechanism of Mut, it is important to analyze the bonding interactions, including HBs and fluorine-mediated halogen interactions. Of particular importance is to determine whether persistent HBs are being formed with the flap-tips in the native-like and P2′-withdrawal binding modes.

### 3.2 Bonding Interactions

#### 3.2.1 Hydrogen Bonding

The establishment of intraprotein HBs within HIV-1 PR contributes to its structural stability and, consequently, to its catalytic activity. In fact, PR active site maintains its assembly through the establishment of a HB network, known as the fireman’s grip,^97^ where each of the T26 residues acts as a critical bridge stabilizing the dimer, by donating HBs to T26 and L24 from the opposing monomer. Thus, to examine the potential role of the mutations in the establishment of these fundamental interactions for Mut activity, we calculated the number of intraprotein and intermonomer HBs in each system. As can be seen in Figure S8a,b in the ESI, the average number of intraprotein HBs does not show an appreciable change between Mut–G and Wt–G (≈145). The same occurs with the average number of intermonomer HBs shown in Figure S8c,d in the ESI (≈23 in both systems). However, the occupancy of HBs needs to be considered, since only high occupancies reflect a significant contribution to the protein’s overall structural stability. As a mean to compare, we employed a previously reported criterion for PI–PR systems, and HBs with occupancies ≥70.00% were considered to be persistent.^75^ Following this criterion, no significant change in the average number of intraprotein HBs is observed between Wt–G (Rep1:100, Rep2:103, average = 101.5) and Mut–G (Rep1:105, Rep2:101, average = 103). When focusing solely on intermonomer HBs (chains A and B in Figure 1), an average of 12 persistent HBs are found in Wt–G (Rep1:10, Rep2:14) compared to an average of 11.5 in Mut–G (Rep1:12, Rep2:11). From the fireman’s grip HBs, those involving bridging between residues T26 of opposing monomers, have average occupancies of 58.2% in Wt–G and 73.3% Mut–G. The HBs involving bridging between T26 and L24 (I 24 in Mut) of opposing monomers have occupancies higher than 90% both in Wt–G and Mut–G systems. Interestingly, the average occupancies are higher in Mut–G (≈95.17) compared to Wt–G (≈89.8%), which points to the ability of Mut–G to preserve important HBs stabilizing the active site region via the mutated residues I24 and I24’. These observations support previous knowledge that the resistance mechanism of Mut does not involve a destabilization of its overall structure so that it can retain its enzymatic activity; thereby, it must predominantly rely on hampering inhibitor binding and ultimately causing its release from the active site.

Accordingly, we concentrated on the distinct interactions between the ligand and the protein in Wt–G and Mut–G complexes, to compare how they diverge. Figure 5a–c shows the dynamical evolution of the average number of drug···protein HBs, for both systems. In Wt–G, the average number of HBs is kept constant at ≈3.0 throughout the MD run. In contrast, in Mut–G, the average number of HBs oscillates between 3 and 4 during the first 250 ns of simulation, and more importantly, exhibits a sharp decrease beyond 400 ns, which is consistent in both replicas. This indicates that the backbone-binding inhibitory mechanism of G in Mut–G begins to be disrupted in this time interval. Our MD extensions to 1 μs for Mut–G were mainly motivated to determine the fate of G under a debilitated network of HB mediating its binding. We wondered whether this disruption could lead to a release of G from the binding cavity, observable within the time scale of our simulations.

**Figure 5:**
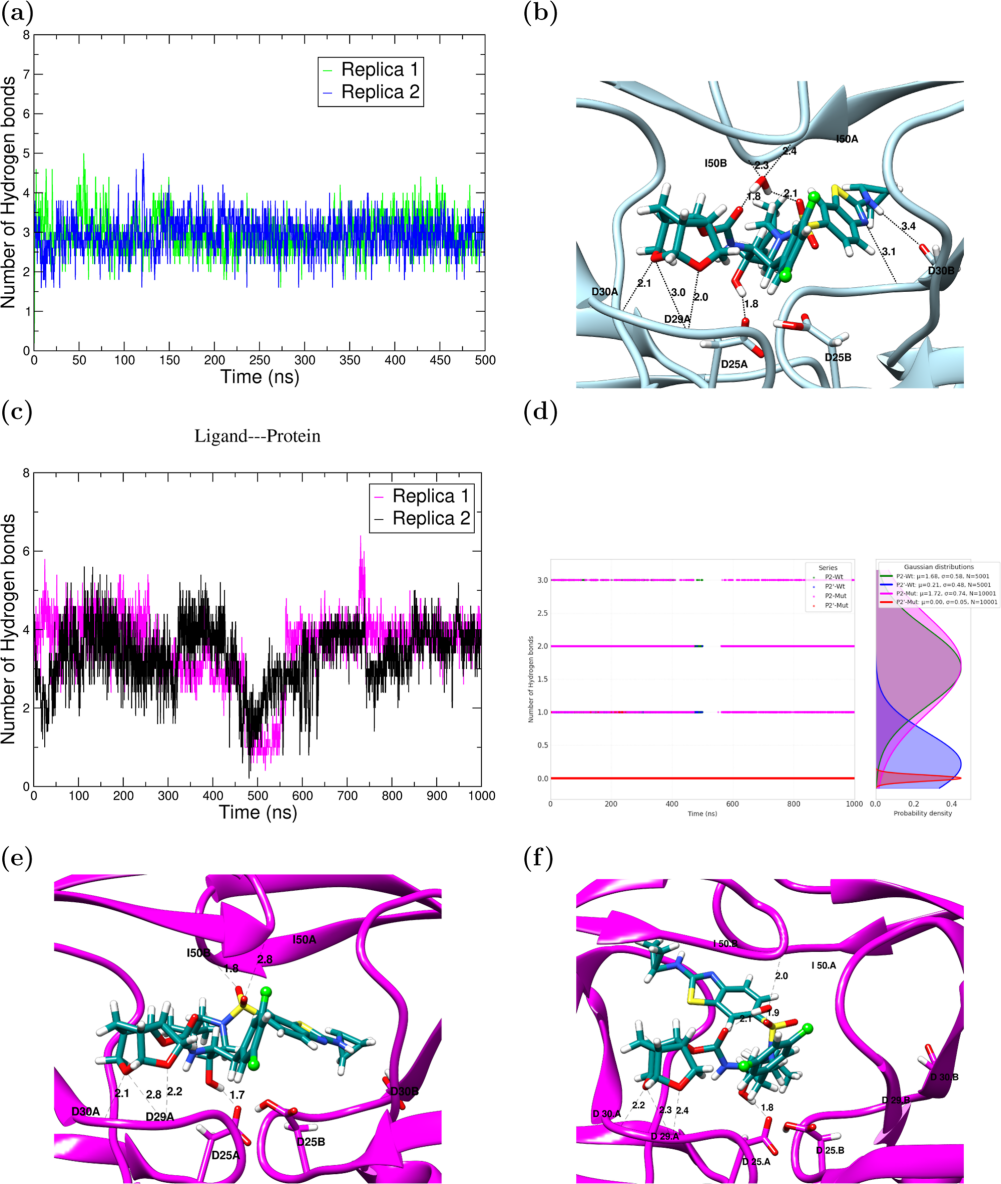
(a) Time evolution of protein–inhibitor HBs in Wt–G complex (b) structure and HBs in the Native binding mode of G in Wt–G (c) time evolution of protein–inhibitor HBs in Mut–G complex. (d) Monitoring of the backbone binding HBs in Wt–G and Mut–G complexes. P2: HBs between P2 moiety (crown-THF) in G and residues D29 A and D30 A. P2′: HBs between P2′ moiety in G and D30 B. (e) Native-like binding mode in Mut–G, exhibiting direct interactions with I50 residues, which ensure a flap-tips closed conformation without flap-water mediation. (f) P2′-withdrawal binding mode in Mut–G, where the interactions with D30 B have been lost.

However, the protein–ligand HBs are regained beyond 500 ns and remain stable. To determine to what extent this restoration comprised the backbone binding HB interactions of P2 and P2′, we closely monitored these HBs during the entire 1 μs run in Figure 5d. (i) HBs between P2 and D29 and D30 of chain A (P2-Wt/P2-Mut). (ii) HBs between P2′ and D30 of chain B (P2′-Wt/P2′-Mut). It is observed that the restoration is mainly driven by the strong backbone binding HBs established by the crown-THF (P2) moiety, while P2′ are no longer contributing. Therefore, we can state that the backbone binding mechanism is not recovered beyond 500 ns due to P2′ permanent detachment. However, this detached P2′-withdrawal conformation also recovers interactions with D25 residues, besides crown-THF, to regain the previous average number of ligand–protein HBs.

We subsequently calculated average occupancies and distances for HBs between PI and PR during each MD replica, for both Wt–G and Mut–G (Tables S2, S3, S4 and S5 in the ESI). As can be observed, the information provided by each Wt–G replica is essentially equivalent. In the case of Mut–G, there are more marked differences in the ranking of HBs by occupancies; however, according to our previous discussion, they are expected given the conformational flexibility of the ligand during the MD run. Consequently, we averaged between replicas to generate a single set of HB distances and HB occupancies to compare the studied systems. This information is presented in Table 2. All the experimentally reported HBs^32,40^ were found during our simulations, including four additional interactions, indicated by asterisks. Visualizations of the main HB interactions between the inhibitor and the protease for the native binding mode in the Wt-G case, and for the native-like and P2′-withdrawal binding modes are displayed in Figure 5b–f.

**Table 2:** Donor (D)–Hydrogen (H)···Acceptor (A) HBs Calculated Using Distance D–A ≤ 3.5 Å and Angle D-H-A ≥ 150°^a^

number	donor	hydrogen	acceptor	G moiety	HB distance	occupancies %
comparison with experimental reports						
1	N D29 A	H D29 A	O5 GRL-142	P2	2.11 (2.14)	91.5 (83.22)
2	N D30 A	H D30 A	O7 GRL-142	P2	2.29 (2.21)	67.8 (75.83)
3	O26 GRL-142	H40 GRL-142	OD2 D25 A	TS binder	1.83 (1.85)	56.6 (48.53)
4	O26 GRL-142	H40 GRL-142	OD1 D25 A	TS binder	1.81 (1.87)	38.75 (*↓*15.92)
5	N45 GRL-142	H38 GRL-142	OD2 D30 B	P2′	2.24 (2.34)	11.37 (*↓*2.77)
6	N45 GRL-142	H38 GRL-142	OD1 D30 B	P2′	2.23 (X)	10.83 (X)
7	N D30 B	H D30 B	N43 GRL-142	P2′	2.39 (2.44)	6.05 (9.2)
8	N D29 A	H D29 A	O7 GRL-142	P2	2.30 (2.29)	4.82 (9.09)
9*	N G48 B	H G48 B	N43 GRL-142	P2′	2.30 (2.35)	3.16 (0.21)
10*	N23 GRL-142	H39 GRL-142	OD2 D25 A	TS binder	2.00 (2.15)	1.85 (0.51)
11*	N I50 B	H I50 B	O35 GRL-142	P2′	2.25 (2.11)	0.72 (*↑*28.29)
12*	N23 GRL-142	H39 GRL-142	OD1 D25 A	TS binder	2.05 (2.17)	0.69 (0.48)
**13**	OD2 D25 B	HD2 D25 B	O26 GRL-142	TS binder	2.02 (X)	0.22 (X)
14	O26 GRL-142	H40 GRL-142	OD1 D25 B	TS binder	2.50 (X)	0.14 (X)
15	N23 GRL-142	H39 GRL-142	O G27 A	TS binder	2.41 (X)	0.02 (X)
**16**	O26 GRL-142	H40 GRL-142	OD2 D25 B	TS binder	2.55 (2.19)	0.02 (3.09*)
interactions unique to Mut-G						
a	N I50 B	H I50 B	O34 GRL-142	P2′	(X) 2.11	(X) 28.29
b	N I50 A	H I50 A	O13 GRL-142	P2	(X) 2.28	(X) 18.8
c	N45 GRL-142	H38 GRL-142	O P79 A	P2′	(X) 2.14	(X) 3.03
d	N I50 B	H I50 B	O35 GRL-142	P2′	(X) 2.31	(X) 2.46
e	N I50 A	H I50 A	O34 GRL-142	P2′	(X) 2.28	(X) 2.26

a Mut–G value is next to wt–g value between parentheses. HB distance: average distance (in Å) between Hydrogen and H-Acceptor. occupancy: average occupancy calculated using 5000 frames (0–500 ns) from the trajectory. HB interactions not reported previously in the literature for Wt–G are indicated by a *. interactions found in the mut–g system, that were not previously reported, are listed at the bottom with letters a-f. Labels for heavy atoms of g can be consulted in Figure 1c.

The occupancy values are complementary to the information in Figure 5, as they allow to identify the main contributors to the average of 3 constant HBs interactions that are held during the entire MD simulation in the case of Wt–G, and the 3 to 4 average HBs during the first 400 ns of simulation, in the case of Mut–G. It can be observed that in both Wt–G and Mut–G, two main interactions are dominant during the entire simulations, namely, HBs involving both oxygens of the crown-THF moiety in G, and D 29 and D 30 in chain A of PR (interactions **1** and **2**). The other one or two HBs contributing to time averages in Figure 5, are most likely those involving interactions between –OH of the transition state binder moiety of G, with both oxygens of D25 in chain A (interactions labeled as **3** and **4** in Table 2 consistently found in both systems). The remaining experimental HBs (from 5 onward) have occupancies that range from moderate to very poor. HB involving the protonated aspartate oxygen OD2 with the −OH (O26) group from the transition state binder was not consistently encountered in both replicas. Rep1 recovered this interaction with OD2 as the HB donor (interaction **13** in Table 2, occupancy = 0.22%) and Rep2 retrieved OD2 as HB acceptor (interaction **16** in Table 2, occupancy = 0.02%).

Interestingly, four HBs were found that have not been previously reported (interactions **9–12**), and whose occupancies are higher than the lowest occupancy computed for the experimental HBs. However, all four bonds are still very poorly occupied. A HB interaction from the amide nitrogen of the G carbamate moiety to the backbone carbonyl oxygen of G27 has been reported in the literature^32^ (HB number **15**). However, this interaction was not consistently found during our MD simulations, it was only found in Rep1 with a distance of 2.41 Å, occupancy 0.02%. As can be observed, the N23 atom in G prefers to interact with both oxygens of D25 in chain A, though with marginal occupancies (interactions **10** and **12**).

No general trend is observed when comparing average HB distances between Mut–G and Wt–G in Table 2. Notwithstanding, in terms of occupancies, it can be seen that the Mut–G complex presents significantly lower occupancies (decrease >10.0% indicated by red arrows) in some of the paramount HBs in Wt–G complex. Aggravatingly, some HBs are completely lost in Mut–G complex (HB numbers **6**, **13–15**). The latter involves the D residues either as HB acceptors or donors. Hence, the resistance mechanisms of Mut involve a disruption of the key HB network that stabilizes the protein–ligand backbone-binding mechanism.

Lastly, interactions that are unique to Mut–G, i.e, they are not present in the Wt–G, are shown at the bottom of Table 2a–e for comparison. Moreover, their occupancies are significant and often exceed, or at least are comparable to, most of the computed occupancies for the experimentally reported HBs in Wt–G. As anticipated by our structural analysis, these interactions involve the flap-tips. Interestingly, the specific atoms in these HBs are those that participate in the flap-water mediated HB network in Wt–G, which promotes ligand binding stability by keeping the flap tips in a closed conformation. Nevertheless, the interactions are not being mediated by the flap-water, but are direct interactions between the Mut PR and the PI. To gain insight into how this important mechanism may vary between Wt–G and Mut–G, next we followed the time evolution of the corresponding HBs.

#### 3.2.2 Flap Water-Mediated Interactions

In addition to direct HB interactions between PI drugs and HIV-1 Pr, there are four crucial HBs in the ligand–flap interface mediated by a conserved water molecule. Experimental X-ray and NMR studies, along with MD simulations, have shown that, with the establishment of these 4 persistent HBs, the flap-water regulates flap-closing dynamics, and modulates ligand binding through enthalpic and entropic contributions.^98^ Our Wt–G simulations are in agreement with the expected stabilizing effect of this flap water. There is a constant number of 4 HBs mediated by the water (Figure S9a in the ESI). A close following of these 4 HBs in Wt–G, reveals that there is always at least one water molecule mediating this HB network. However, this stabilization is not due to the contribution of a unique water molecule; rather, waters switch positions throughout MD simulations to mediate between the ligand and the flap-tips, ensuring flap-closed conformation in time, with the maximum time occupancies for a flap water being 8.3% and 25.2% in Rep1 and Rep2, respectively. By contrast, the corresponding flap-water mediated HBs in Mut–G are disrupted early in the simulation and, although partially recovered, remain unstable throughout the 1 μs MD run (Figures S9b and S2d in the ESI). These observations are in line with previous studies that have reported a debilitation of the flap-water mediation between inhibitors and mutated proteases.^99^ However, despite the early loss of this flap-water interaction, the flaps do not open during the entire 1 μs simulation. As can be observed in Figures 5e and S9b in the ESI, the closed conformation of the flaps is retained via direct HBs between the ligand group sulfone oxygens and the main chain N–H groups of I50 and I50’. These HBs are unique to Mut–G and take place when the water-mediated ones have been disrupted. Furthermore, these flap water HBs, along with those mediated by the crown-THF moiety, are responsible for the restoration of the average number of HBs in the protein···ligand complex beyond 500 ns.

#### 3.2.3 Fluorine-Mediated Halogen Interactions

One central difference between DRV and G lies in fluorine. The original P1 phenylmethyl group of DRV has been replaced by a difluorophenylmethyl moiety in G. Fluorine substitutions in this modified P1 group have been suggested to confer higher CNS penetration, as well as to improve binding affinity through the establishment of novel interactions with the active site residues. Pietruś et al.^100^ have recently reported that the contact F···H–X, has characteristics of a HB with angles and distances falling within the atypical ranges 150°–120° and 2.9–3.6 Å, respectively. The most common type of interaction favors hydrophobic environments by establishing F···H–C contacts, but there also exist F···H–N interactions, usually involving amino groups in side chains. Notably, these interactions depend only on the donor–acceptor distance, and not on the angle F–H–X, due to the nearly uniform spatial distribution of the three free electron pairs in fluorine.^100^ After applying the former thresholds for angle and distance, Figure S10 in the ESI shows that the number of fluorine-mediated HBs between G and the protein remains approximately constant at ≈5 in both Wt–G and Mut–G complexes.

The occupancies of the established interactions with the corresponding residue, and their minimum average F···H–D distances throughout the trajectories (from either the main and/or side chain hydrogens), are listed in Table 3. These interactions are present in two predominant protease regions: the triad P81–V82–I84 in Wt (P81–I82–V84 in Mut) and the conserved region G49-I50. Figure 6 shows such interactions in exemplary frames from the MD of Wt–G and Mut–G. Individual residues A28 and R8 were also common in both complexes. Furthermore, the non-native Fluorine-I32 interaction appeared in Mut–G (Figure 6c). Remarkably, G is capable of conserving these fluorine interactions during the entire simulation (see Figure S10), including interactions with mutants I82 and V84, despite P2′ having already abandoned its pocket. This highlights the contribution of fluorine substitutions to the inhibitor antiviral potency in combating resistant strains. These contacts must also contribute to the improved efficiency of G over DRV, given that the latter lacks these extra interactions.

**Table 3:** Donor (D)–Hydrogen (H)···Fluorine (F) Interactions Calculated Using Cutoff Distance D-F ≤ 4.6 Å and Angle D–H–F ≥ 120°^a^

residue	occupancy (%)	distance	residue	occupancy (%)	distance	type
involved	in Wt-G		involved	in Mut-G		
P81 side	128 (87)	2.84 (3.26)	P81 side	121 (119)	3.08 (2.96)	C–H···F
**V82** side	109 (85)	3.17 (12.10)	**I82** side	97 (89)	3.19 (3.14)	C–H···F
I50 side	86 (60)	2.98 (3.04)	I50 side	91 (65)	3.01 (3.50)	C–H···F
**I84** side	69 (81)	3.44 (3.29)	**V84** side	34 (14)	4.17 (5.07)	C–H···F
G49 main	52 (14)	3.17 (3.86)	G49 main	37 (43)	3.76 (3.86)	C–H···F
L23 side	28 (28)	2.98 (2.87)	L23 side	22 (22)	3.56 (3.53)	C–H···F
I50 main	13 (45)	3.43 (3.24)	I50 main	8 (4)	3.92 (4.34)	N–H···F
R8 side	13 (18)	3.64 (3.37)	R8 side	16 (27)	4.32 (4.03)	N–H···F
						C–H···F
A28 main	9 (33)	4.6 (4.0)	A28 main	2 (24)	5.43 (4.42)	C–H···F
			**I32** side	10 (7)	6.38 (7.54)	C–H···F

a Value in Rep2 is next to Rep1 value between parentheses. Main: main backbone residue atoms. Side: Side Chain Residue atoms. Average distances are reported in Å.

**Figure 6:**
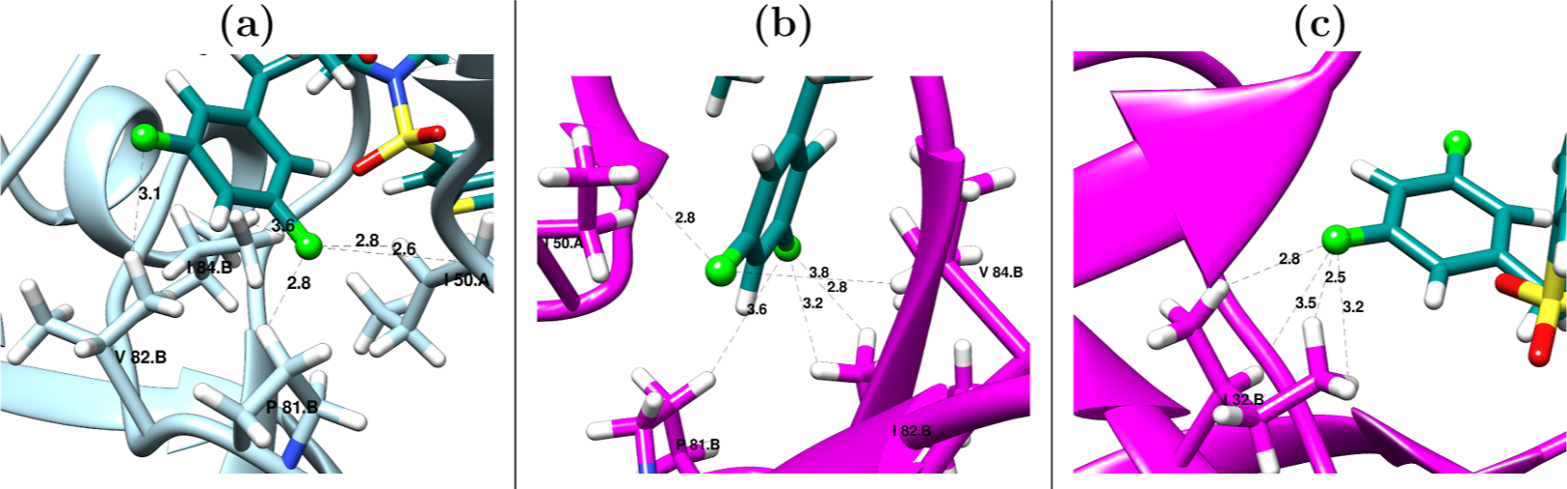
Fluorine interactions at 500 ns. (a) Wt–G Rep1 (b) Mut–G Rep1 (c) Longer V32I side-chain allows non-native contacts with Fluorine. All distances in Å.

#### 3.2.4 Effect of L33F and I54M Mutations on Differential Hydrophobic Interactions

Experiments previously revealed that the exceptional subnanomolar antiviral activity of G against Wt is preserved in the presence of the highly DRV-resistant HIV-1 variant p30, and is even improved against the resistant variant p20. Our Mut model, p51, contains all mutations that are present in both p30 and p20, plus the two additional mutations L33F and I54M, resulting in up to 436-fold decrease in G antiviral activity. Thus, we closely monitored the interactions established by residues 33 and 54 in Wt–G and Mut–G in order to evaluate the role of the mutant residues in the mechanism of resistance. Both amino acids are nonpolar, and may engage in hydrophobic interactions. To follow the hydrophobic interactions, we applied a distance cutoff of 4.5 Å between the heavy atoms of G and each of the residues in Wt–G and Mut–G, which has been previously reported to capture the hydrophobic interactions in protein–ligand systems based on analyses of a series of PDB structures.^101^ As shown in Figure S11a,b in the ESI, there are no changes in the minimum distances involving residues L33 and F33, in their respective complexes, indicating an absence of contribution to hydrophobic interactions and suggesting a conservative nature for the L33F mutation. In the case of I54M mutation (Figure S11d,e in the ESI), based on the short distances (≈3.5 Å) and the number of contacts observed during the simulation, we conclude that hydrophobic interactions are established between M54 and G in Mut-G complex. These interactions are absent in Wt–G and help stabilize P2′-withdrawal conformations in Mut–G, which is indicative of a nonconservative nature for the I54 M mutation.

### 3.3 Energetics of Protease···G Binding via MMPBSA Calculations

To address the energetics of protein···ligand binding, MMPBSA calculations were performed on both Wt–G and Mut–G systems. The details of the results for each system replica, including energy decomposition, can be consulted in Table 4. The binding energies for Wt–G were calculated to be Δ*G*_Wt_Rep1_ = −15.94 kcal/mol and Δ*G*_Wt_Rep2_ = −16.19 kcal/mol, which are in good agreement with the experimental value of Δ*G* = −14.9 kcal/mol.^25,102^ For Mut–G, the corresponding values were Δ*G*_Mut_Rep1_ = −15.45 kcal/mol and Δ*G*_Mut_Rep2_ = −19.59 kcal/mol. In the case of Rep3, the total binding energy during the 1 μs trajectory was Δ*G*_Mut_Rep3_ = −11.32 kcal/mol (Table S8 in ESI), thereby, resulting in an average for the Mut–G binding energy of −15.45 kcal/mol, compared to −16.07 kcal/mol for the Wt–G system. Thus, Wt–G shows a slightly better binding affinity than Mut–G, yet, the binding energies of both systems remain comparable. Accordingly, to better understand these findings, which might be puzzling at first glance, we first examine some details of the MMPBSA binding energies, including energy contributions and total energy values across the different structural intervals considered.

**Table 4:** Binding Free Energies for Wt–G and Mut–G Computed with MMPBSA^e^

energy	Wt–G		Mut–G	
contribution^a^	Rep1	Rep2	Rep1	Rep2
Δ*E*_vdw_	–75.14(5.05)^b^(0.36)^c^	–77.98(4.25)(0.30)	–73.86(5.18)(0.26)	–76.93(5.83)(0.29)
Δ*E*_ele_	–28.37(6.17)(0.44)	–32.49(7.04)(0.50)	–26.32(5.58)(0.28)	–26.48(6.02)(0.30)
Δ*G*_pol_	61.37(6.96)(0.49)	65.47(7.10)(0.50)	58.46(6.28)(0.31)	58.21(6.41)(0.32)
Δ*G*_nonpol_	–6.61(0.17)(0.01)	–6.65(0.17)(0.01)	–6.71(0.20)(0.01)	–6.74(0.17)(0.01)
Δ*H*	–48.73(4.53)(0.32)	–51.65(4.41)(0.31)	–48.43(5.65)(0.28)	–50.94(6.57)(0.33)
–*T*Δ*S*	32.79(6.90)(0.49)	35.47(5.37)(0.38)	33.00(5.50)(0.27)	31.35(6.37)(0.32)
Δ*G*	–15.94(8.24)(0.59)	–16.19(6.94)(0.50)	–15.45(7.76)(0.39)	–19.59(8.94)(0.45)
Δ*G*_exp_	–14.9^d^		N/A	

a Wt–G binding energies were computed through the 500 ns trajectories. Mut–G binding energies were computed throughout the entire 1 μs trajectory.

b Standard deviation (SD).

c Standard error of the mean (SEM).

d Wt–G experimental binding energy was obtained using Δ*G*_exp_ = *RT* ln *K*_*i*_ with the reported inhibition constant *K*_*i*_ ^25^.

e All energies are reported in kcal/mol. Entropy corrections were carried out via normal mode analysis.

In general, the energetic contributions to the binding in all systems are consistent. The main contributor to the binding energy is the Δ*E*_vdw_ term, which ranges from −78 to −74 kcal/mol, followed by Δ*E*_ele_ term, ranging from −32 to −26 kcal/mol. All the nonpolar contributions to the solvation energies exhibit a relatively stabilizing effect, though less, compared to the former terms (≈ −7 kcal/mol). Conversely, the Δ*G*_pol_ term is destabilizing, representing a cost that ranges from 59 to 65 kcal/mol across the systems. There is also an entropic penalty (−*T*Δ*S*) to binding within 31–35 kcal/mol. It must be noted that only with the estimation of this entropic penalty contribution, values that are close to the experimentally measured ones can be obtained. A most noticeable difference between systems, is the consistent higher positive value for the polar solvation term in Wt–G compared to Mut–G (at least 3 kcal/mol gap), which is consistent with a more buried conformation of the ligand in Wt, compared to Mut, in which polar groups in P2′ are more exposed to the solvent, resulting in a less unfavorable contribution of Δ*G*_pol_.

When comparing between Wt–G and Mut–G systems, it is observed that Wt–G exhibits more consistent favorable contributions from Δ*E*_vdw_ and Δ*E*_ele_, except for the nonpolar contribution to solvation which is slightly larger in magnitude for Mut–G (−6.71 vs –6.61 kcal/mol).

We now turn to an analysis of the energetics of binding in Mut–G during the first 500 ns simulations. To estimate the binding energy of each binding mode, i.e, native-like and P2′-withdrawal, we calculated weighted averages based on the binding energy of each region, shown in Table 1. The details of the calculations, including weighted averages per group and main binding modes, can be consulted in Tables S6 and S7 in the ESI. In each replica, the native-like binding mode exhibited a higher binding affinity compared to the P2′-withdrawal (−17.2 vs −12.6 kcal/mol in Rep1 and −23.2 vs –21.2 in Rep2). It can be observed that the final regions of each replica (R5 and R6) exhibit the smaller values of binding energies, consistent with a debilitation of the backbone binding HB network at the end of the 500 ns simulations. Regardless of this behavior, the striking recovery of the protein···ligand interactions (Figure S2a in ESI) in both replicas is in line with a binding mode transition to a novel conformation of G (Figure 4 and Region R8 in Figure S1b in the ESI) with marked high affinities of Δ*G*_Mut_1_R8_ = −17.2 kcal/mol and Δ*G*_Mut_2_R8_ = −19.6, higher than Wt–G. This final conformation in R8 constitutes a stabilization of the conformation already explored in R6 of Rep1 and contributes significantly to the average binding energy previously reported for the entire 1 μs simulation.

The previous purely energetic considerations, however, should not obscure the main goal behind the design of G, which consists of a mimesis of the natural HIV-1 PR substrate through precise pharmacophoric interactions with the active site. G is the ultimate drug optimization for excelling in the backbone binding mechanism. Our MD simulations indicate that this mechanism is stable in Wt–G but is partially lost in p51, specifically in the P2′ moiety of G. The transient sampling of regions with unfavorable enthalpy and loss of binding interactions (Table 1 and Figure S2a at around 500 ns), as well as the energetic stability of the novel half-detached conformation in R8, must be interpreted in the context of competitive inhibition with the natural substrate. These results indicate a destabilization of the bioactive conformation of G in p51, which may render it less competitive, contributing to differences in IC50 values. Thus, we argue that the debilitation of the backbone binding mechanism of G against p51 provides a molecular basis for the reported reduction in its antiviral potency.

This final flap-tip conformation in R8 represents an interesting energetic result and may be relevant for future studies exploring the potentialities of inhibitor G. Indeed, a similar conformation of G, which retains interactions at P2 and P1, but involves a reorientation of P2′, has recently been shown to inhibit the HIV-1 integrase enzyme.^41^ To the best of our knowledge, this binding mode of G in p51 has not been previously reported. Thus, from a structure-based drug design perspective, beyond highlighting this interesting binding mode of G, our findings indicate that its P2′ moiety must be optimized to retain the backbone binding inhibitory mechanism against the multi-PI inhibitor resistant strain p51.

## 4 Conclusions

This work has used all-atom molecular dynamics simulations to address the resistance mechanism of a highly resistant HIV-1 PR against the novel, central nervous system penetrating, protease inhibitor G. We compared the time-evolution, interactions, and binding energetics of complexes between G and two model HIV-1 proteases: a wild-type protease and a highly resistant protease, p51. As expected, the backbone-binding mechanism, which involves interactions of the P2 and P2′ moieties with protease subsites S2 and S2′, is observed in Wt–G. However, in Mut–G, the ligand does not reach the stable, bioactive conformation observed in Wt–G, but rather it explores various conformations, even early in the simulations, that can be grouped into two main binding modes referred herein as native-like and P2′-withdrawal binding modes. The native-like resembles the backbone-binded conformation responsible for the inhibitory activity of G against Wild-type PR, while the P2′-withdrawal implies a critical detachment of the P2′ moiety. Even though, both modes are to the detriment of the backbone binding network to face resistance. Our findings indicate an expansion of the active site cavity in the Mut–G complex compared to Wt–G complex, which does not result in an observable opening of the flap-tips. In addition, the flap-water mediated network that modulates closing of the flaps in Wt–G, is disrupted most of the time. The maintenance of the flap-tips closed conformation can be ascribed to their direct interactions with the sulfone group of the ligand that compensate for the absence of flap-water. These are unique interactions in Mut–G complex. The P1′ moiety is a highly mobile group both in Wt–G and Mut–G. Regarding the P1 moiety, its fluorine interactions, mostly of the type C–H···F, are well preserved in Mut–G, and even additional interactions that are unique to Mut–G are formed. This suggests the prominent role of fluorine in binding, not only with Wt but also with a highly resistant protease. It must be stressed that the previous structural fluctuations in G, pointing to its versatility and highlighting novel binding modes, have not been previously reported, likely because they can only be captured with long MD simulations that adequately sample the motion of the protein domains. To the best of our knowledge, the only previous MD study addressing G has covered up to 100 ns.^75^

Overall, our findings allow us to rationalize a resistant mechanism in Mut protease which involves an expansion of its active site cavity, disruption of the native key ligand···protein hydrogen bonding network, and a high structural fluctuation of the ligand that leads to conformations distinct from the bioactive. Accordingly, G reaccommodates in an expanded active site cavity, and ultimately promotes, structurally and energetically, a novel binding mode in which the backbone-binding pharmacophoric interactions that define the bioactive conformation are lost, despite having a final binding energy comparable to that of Wt–G. Thus, from a structure-based drug design perspective, this emphasizes the potency of G to conformationally adapt to resistant proteases and could also be relevant in the design of improved inhibitors. The P1 and P2 moieties are desirable groups in G to be retained, given the persistence of their interactions. P1′ could be further improved given its high mobility. Most importantly, the P2′ fragment is the clear candidate for further optimization of G to combat p51 drug resistance.

## Supplementary Material



## Data Availability

Statement: The crystal structures used in this study were downloaded from the Protein Data Bank (https://www.rcsb.org/, PDB ID: 5TYS and 6MKL). Cartesian coordinates (gro format) for Wt-G and Mut-G complexes used to start production stages in the MD runs, the parameters and topology files for the GROMACS simulations are deposited in Zenodo (DOI: 10.5281/zenodo.17487473).

## Notes

It includes details about the parameters used for docking calculations. Moreover, RMSD, RMSF, radius of gyration, and distance criteria D1 and D3 for the 500 ns Wt—G and 1 μs Mut—G simulations can be found. Computed active-site volumes and structural superpositions of Wt—G and Mut—G at key snapshots in the simulation are also provided. A complementary HB analysis is presented, which covers the time-evolution of intraprotein, intermonomer and flap-water HBs, as well as per-replica tables of HB distances and occupancies. The time-evolution of fluorine-mediated interactions and hydrophobic interactions can be consulted. Additionally, tables summarizing total MMPBSA energies by region, group, and binding mode are included. A section for the Rep3 with details of binding energy calculations and ligand binding poses is presented. Finally, a section with results from the MD runs of G in pure water has been included (PDF)
