# Supplementary material for: Ligand Versatility and Resistance Mechanism of Monotherapy-Grade HIV‑1 Protease Inhibitor GRL-142 Binding the Multidrug Resistant Variant p51: Insights from 1 μs MD Simulations
Source: J Chem Inf Model. 2026 Feb 27;66(6):3220–36. doi: 10.1021/acs.jcim.5c02652 (PMC13014452; doi:10.1021/acs.jcim.5c02652)
Supplement: Supplementary file 1 [file ci5c02652_si_001.pdf]

Ligand Versatility and Resistance Mechanism of Monotherapy-grade HIV-1 Protease Inhibitor GRL-142 Binding the Multidrug Resistant Variant p51: Insights from 1 μs MD Simulations

Supporting Information

Alejandro Arias,[†] Chiara Cappelli,[‡] Albeiro Restrepo[†]
Jorge Alí-Torres,[¶] and Sara Gómez,[¶]

[†]*Instituto de Química, Universidad de Antioquia UdeA, Calle 70 No. 52-21, Medellín, Colombia*
[‡]*Scuola Normale Superiore, Classe di Scienze, Piazza dei Cavalieri 7, 56126, Pisa, Italy* [¶]*Departamento de Química, Universidad Nacional de Colombia, Sede Bogotá*

E-mail: sagomezam@unal.edu.co

Contents

1 Molecular docking simulations S2
1.1 Docking details . . . S2
1.2 Docking Results . . . S2

2 Mut–G simulations: Extension to 1 μs S3

3 Structural stability during 0-500 ns interval S5
3.1 Protease . . . S5
3.2 Inhibitor GRL-142 . . . S7

4 Interactions taking place during 0-500 ns interval S8
4.1 Protein-Protein . . . S8
4.2 Protein-Inhibitor . . . S9
4.2.1 Direct interactions . . . S9
4.2.2 Water-mediated interactions . . . S13
4.2.3 Fluorine-mediated interactions . . . S14
4.2.4 Effect of specific mutations L33F and I54M . . . S15

5 MMPBSA binding energies S16
5.1 Total energies by region, group and binding mode . . . S16

6 Analysis of the results of Replica 3 for Mut–G S17

7 MD simulations of GRL-142 in pure water S18

1 Molecular docking simulations

1.1 Docking details

For the dockings with Swiss-dock[1, 2], the Attracting-cavities (AC) method was utilized. For Wt–G, the following parameters were used: Sampling exhaustivity=90, cavity prioritization=70, Random initial conditions=1, Box size = (15, 15, 27) and Box center= (-9.0, 15.0, 0). For Mut–G, the corresponding parameters were 90, 70, 1, (15, 15, 27) and (9, -15, 0). The RMSD with respect to the crystallographic pose was 0.93 Å for Wt–G and 1.15 Å for Mut–G.

For docking with Autodock Vina [3, 4], the following parameters were applied for both Wt–G and Mut–G: energy_range = 3, exhaustiveness = 20, num_modes = 20 and box size = (15, 15, 27). G was placed at coordinates (-9, 15, 0) for Wt–G and at (9, -15, 0) for Mut–G. The RMSD value with respect to the crystal ligand was 0.67 Åfor Wt–G and 0.76 for Mut–G.

Based on the RMSD values found in each program, which are consistently below 2.0 Å, both programs were able to reproduce the crystal binding pose of the ligand in their corresponding receptors (PDB: 5TYS, PDB: 6MKL). Moreover the hydrogen bond network established by the crystal water was also reproduced. Therefore, these protocols are reliable protocols to determine an optimal starting pose of the ligand in the receptor active site, and can be used in future studies addressing novel ligands based on the GRL-142 scaffold, for which experimental structure is not available.

1.2 Docking Results

The docking results were able to reproduce the poses that ligand G exhibits both in the Wt-G and Mut-G crystal structures. Although docking results have well-known limitations discussed elsewhere[5, 6, 7], they are cheap methodologies to obtain estimates for ligand$\cdots$protein affinities. Our docking scores approximately reflect the experimental decrease in binding affinity when going from the Wt–G to the Mut–G systems (-10.6 vs -10.4 kcal/mol in Vina, and -9.82 vs -9.74 kcal/mol in Swissdock). To complement this argument, we conducted docking calculations with the current clinically used DRV, obtaining the same trends between Wt–DRV and Mut–DRV (-9.2 vs -8.7 kcal/mol and -10.3 vs -9.5 kcal/mol). Furthermore, these docking studies also support the experimental binding affinities that suggest a slightly improved G potency over DRV ($K_i = 14$ pM, IC50 of 0.017 nM, -14.9 kcal/mol for Wt–G vs -14.8 kcal/mol for Wt–DRV at 300K)[8, 9]. Thus, beyond these rough binding energies, docking results served to determine optimal initial configurations for the Wt-G and Mut-G complexes to start our MD runs.

2 Mut–G simulations: Extension to 1 μs

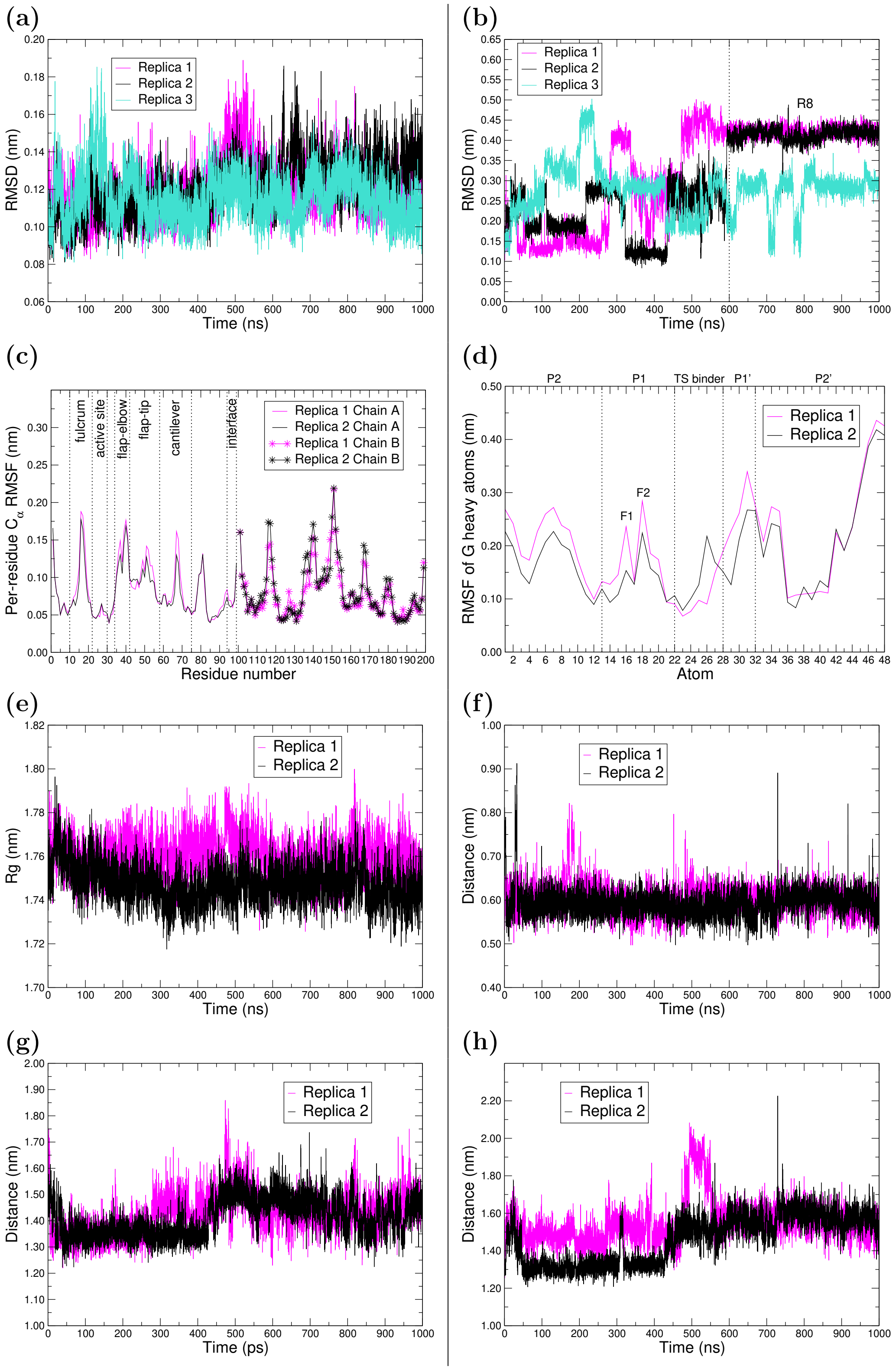

Figure S1: Conformational analysis along the MD run for Mut–G during 1μs. (a) RMSD of Mut backbone atoms. (b) RMSD of ligand G in Mut–G. The converged region R8 is referred solely to Rep1 and Rep2. (c) RMSF of Mut C_α atoms. (d) RMSF of ligand G heavy atoms. (e) Mut radius of gyration. (f) Criterion for the flap-tips conformations D1(I50 A – I50 B). (g-h) Distance criteria for active site volume D2 (D25 A – I50 A) and D3 (D25 B – I50 B).

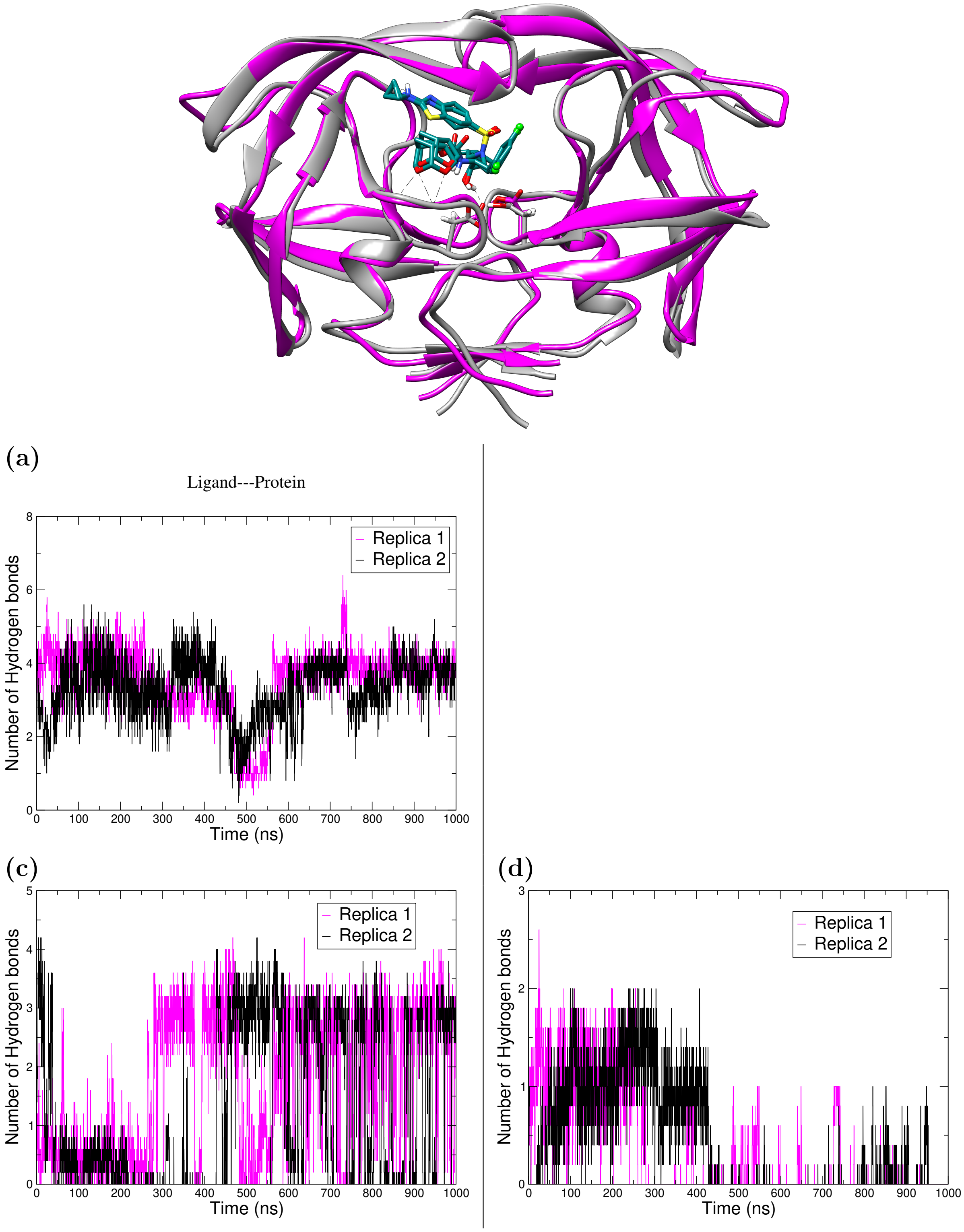

Figure S2: (Top) Superposition of the final binding modes of G at 1μs in Rep1 and Rep2 of Mut–G system. (a) Time evolution of ligand$\cdots$protein HBs (b) Native flap-water Hydrogen bond network in Mut–G, showing early disruption. (c) Direct ligand$\cdots$flap-tips interactions (residues I50A and I50B), which compensate for flap-water loss to keep closed-conformation of flap tips.

3 Structural stability during 0-500 ns interval

3.1 Protease

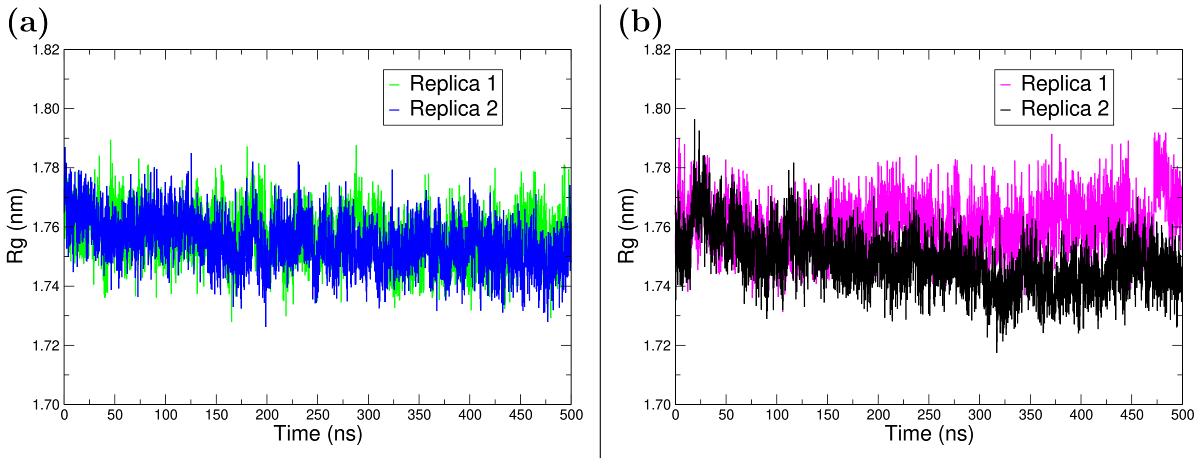

Figure S3: Radius of gyration along the MD run for Wt (a) and Mut (b) in their corresponding complexes with G.

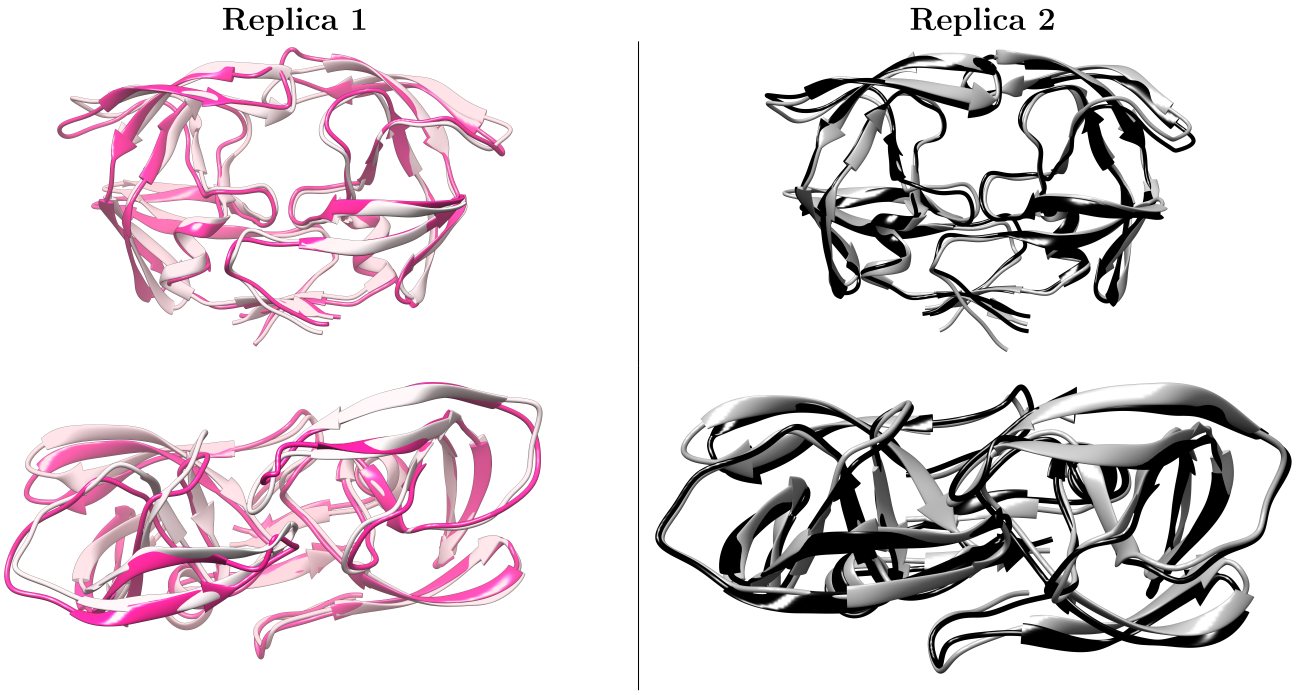

Figure S4: Superpositions of Mut-pr-G at 450 ns (lighter colors) and 500 ns (darker colors). Top: Front view. Bottom: Top-view .

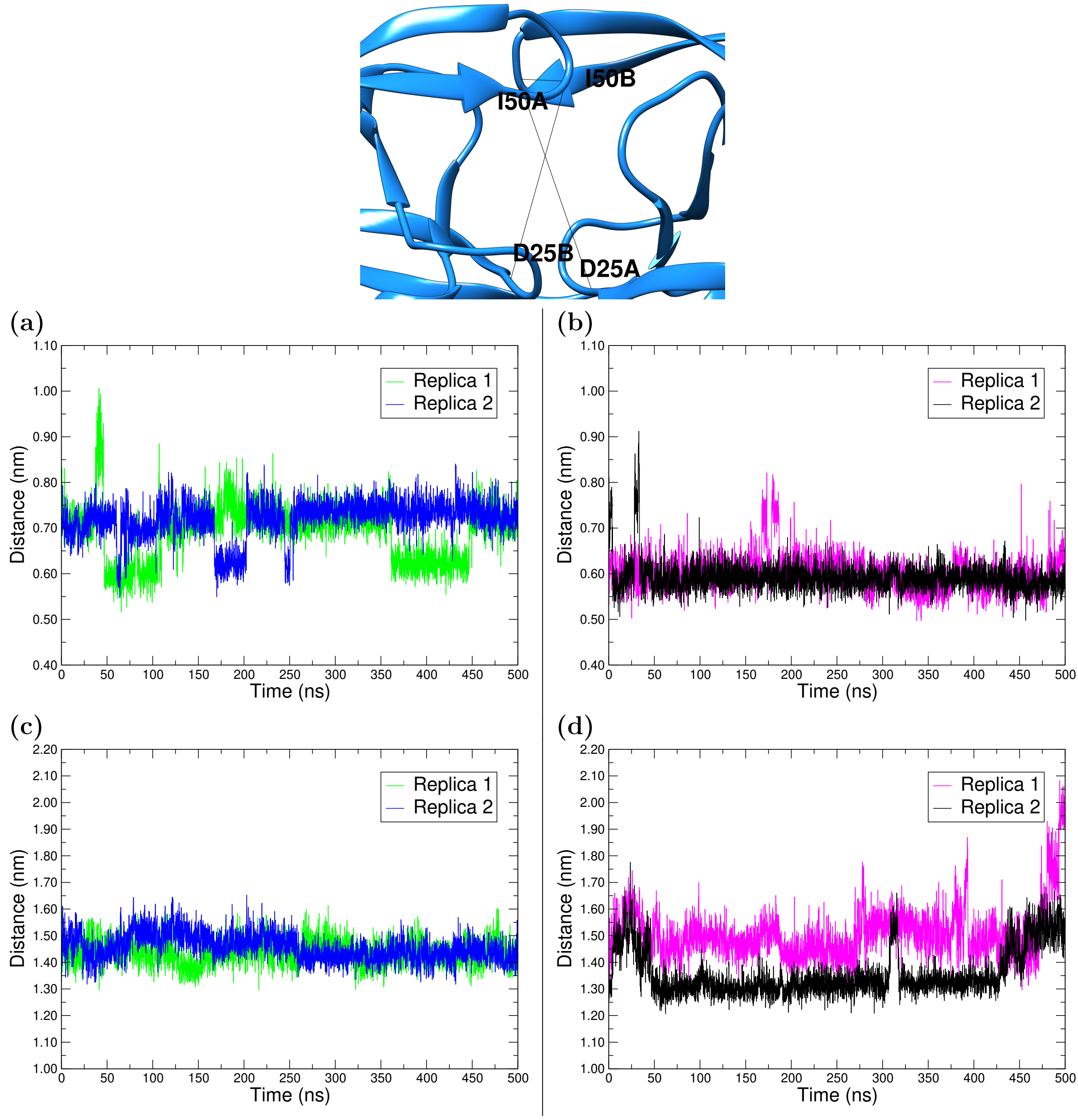

Figure S5: (Top) Detail of the protease active site cavity showing the residues involved in the flap-tip conformations criteria. (a-b) Distance D1 (I50 A – I50 B) criterion for conformations of the flap-tips in Wt–G and Mut–G. (c-d) Distance criterion D3 (D25 B – I50 B) for active site volume.

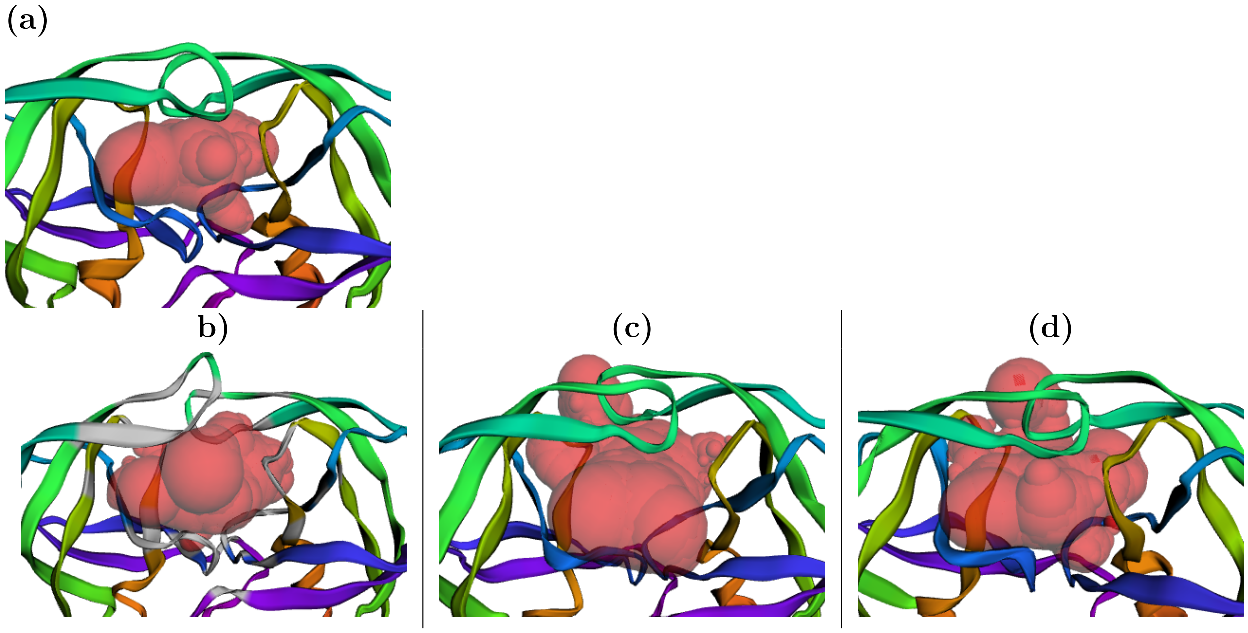

Figure S6: Volume expansion of the Mut-pr-G active site compared to Wt–G. (a) Wt–G Rep1 active site volume = 475.5 Å^3 at 500 ns. (b) Mut–G Rep1 active site volume = 695.7 Å^3 at 500 ns. (c) Mut–G Rep1 active site volume= 885.4 Å^3 at 800 ns. (d) Final active site volume= 644.3 Å^3 in Rep1 at 1 μs.

3.2 Inhibitor GRL-142

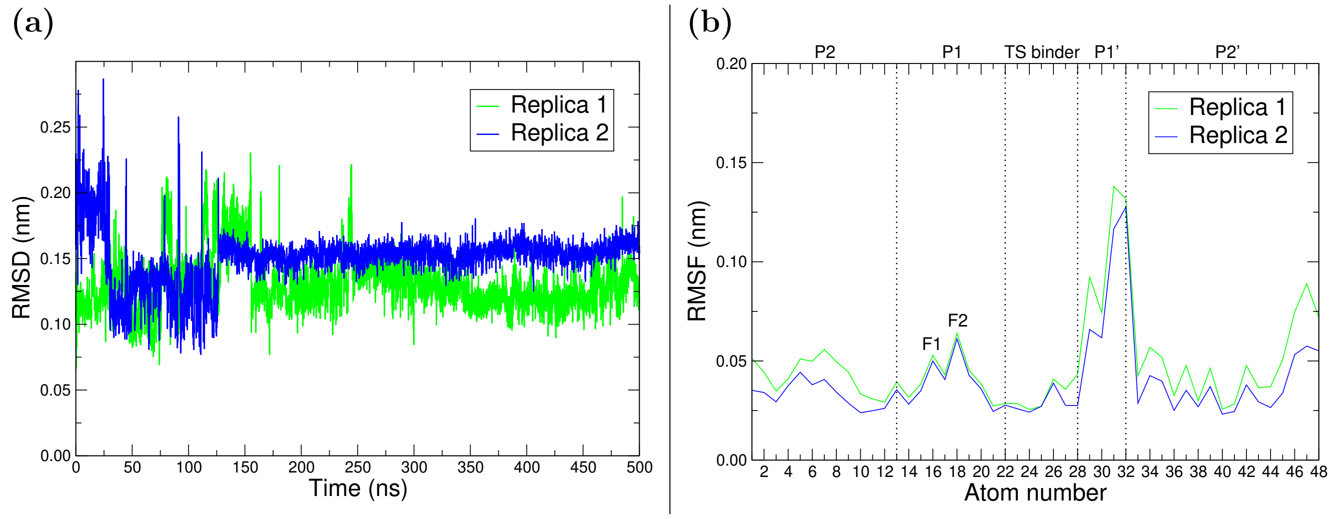

Figure S7: Conformational analysis of ligand G along Wt–G simulation. (a) Ligand RMSD. (b) Ligand RMSF was computed in the interval [150-500] ns.

Table S1: Ligand RMSD values during the 0–500ns trajectories.

System	Replica	RMSD (nm)	SD (nm)
Wt–G	1	0.1307	0.0210
	2	0.1505	0.0201
Mut–G	1	0.2282	0.1004
	2	0.2070	0.0617
	3	0.2850	0.0715

4 Interactions taking place during 0-500 ns interval

4.1 Protein-Protein

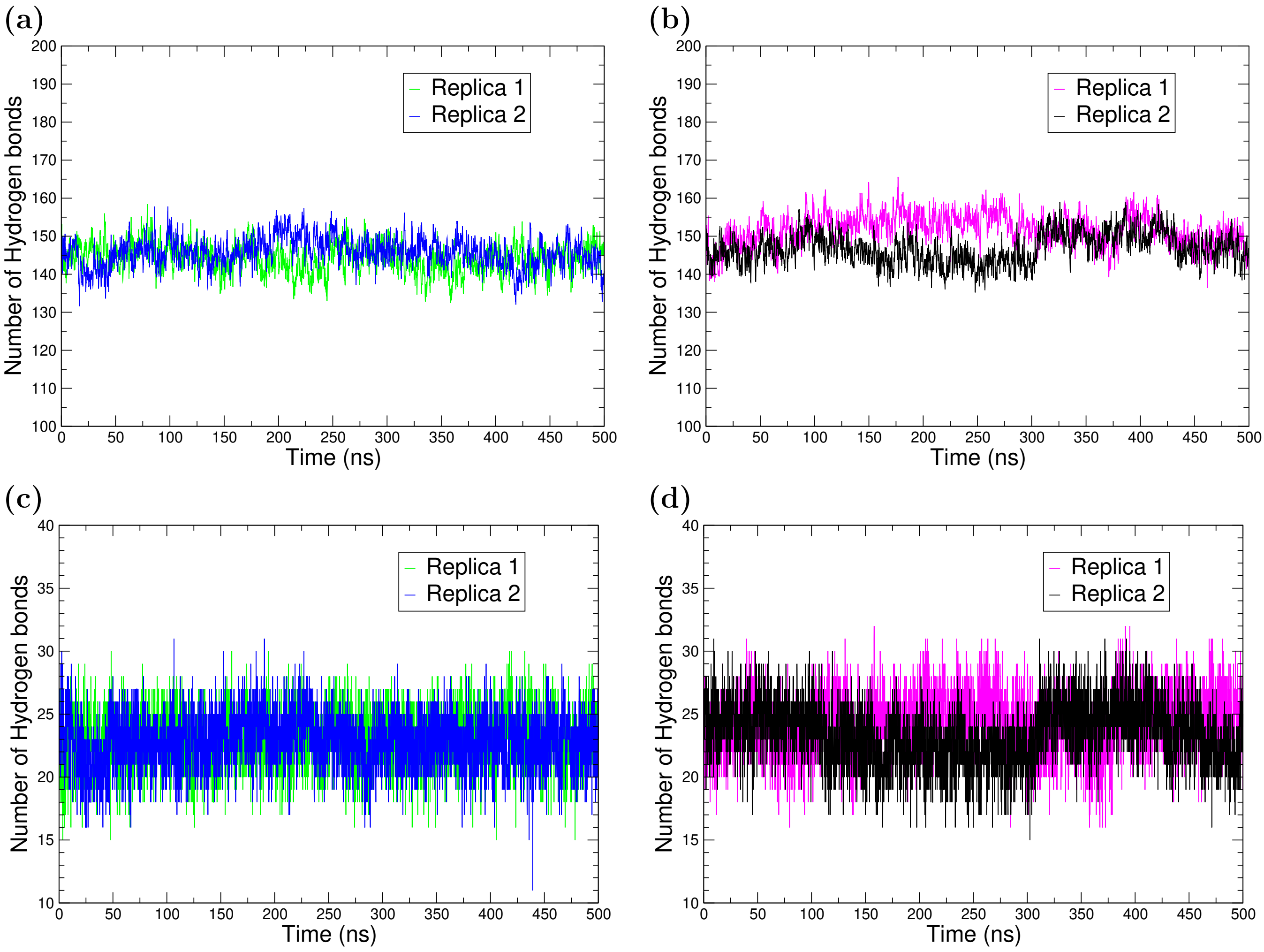

Figure S8: Average number of Intra-protein HBs in Wt–G and Mut–G (a-b). Average number of Inter-monomer HBs in Wt–G and Mut–G. Chains are depicted in Figure 1 (a).

4.2 Protein-Inhibitor

4.2.1 Direct interactions

Table S2: Hydrogen bonds Donor (D)–Hydrogen (H)$\cdots$Acceptor (A) between G and Wt in **Rep1** calculated using distance D-A$\leq$ 3.5 Å and angle D-H-A $\geq$ 150°. HB distance: average distance between Hydrogen and H-Acceptor. Occupancy: average occupancy calculated using 5000 frames from the trajectory. All distances in (Å). G labels in Figure 1.

Number	Donor	Hydrogen	Acceptor	HB distance	Occupancy (%)
1	N D29 A	H D29 A	O5 GRL-142	2.10	93.24
2	N D30 A	H D30 A	O7 GRL-142	2.29	68.25
3	O26 GRL-142	H40 GRL-142	OD1 D25 A	1.76	50.87
4	O26 GRL-142	H40 GRL-142	OD2 D25 A	1.82	46.23
5	N D30 B	H D30 B	N1 GRL-142	2.39	10.24
6	N D29 A	H D29 A	O7 GRL-142	2.30	6.12
7	N45 GRL-142	H38 GRL-142	OD2 D30 B	2.28	5.86
8	N G48 B	H G48 B	N1 GRL-142	2.28	5.42
9	N45 GRL-142	H38 GRL-142	OD1 D30 B	2.25	5.30
10	N23 GRL-142	H39 GRL-142	OD2 D25 A	1.97	1.98
11	N I50 B	H I50 B	O34 GRL-142	2.41	0.68
12	N G48 B	H G48 B	N45 GRL-142	2.43	0.62
13	OD2 D25 B	HD2 D25 B	O26 GRL-142	2.02	0.22
14	N23 GRL-142	H39 GRL-142	OD1 D25 A	2.09	0.14
15	N D29 B	H D29 B	N1 GRL-142	2.54	0.08
16	N I50 A	H I50 A	O34 GRL-142	2.33	0.06
17	O26 GRL-142	H40 GRL-142	OD1 D25 B	2.66	0.04
18	N D30 A	H D30 A	O5 GRL-142	2.20	0.04
19	N45 GRL-142	H38 GRL-142	O M46 B	2.30	0.02
20	N23 GRL-142	H39 GRL-142	O G27 A	2.41	0.02
21	N A28 A	H A28 A	O26 GRL-142	2.40	0.02

Table S3: Hydrogen bonds Donor (D)–Hydrogen (H)$\cdots$Acceptor (A) between G and Wt in **Rep2** calculated using distance D-A$\leq$ 3.5 Å and angle D-H-A $\geq$ 150°. HB distance: average distance between Hydrogen and H-Acceptor. Occupancy: average occupancy calculated using 5000 frames from the trajectory. All distances in (Å). G labels in Figure 1.

Number	Donor	Hydrogen	Acceptor	HB distance	Occupancy (%)
1	N D29 A	H D29 A	O5 GRL-142	2.12	89.76
2	N D30 A	H D30 A	O7 GRL-142	2.28	67.27
3	O26 GRL-142	H40 GRL-142	OD2 D25 A	1.84	66.97
4	O26 GRL-142	H40 GRL-142	OD1 D25 A	1.85	26.63
5	N45 GRL-142	H38 GRL-142	OD2 D30 B	2.20	16.88
6	N45 GRL-142	H38 GRL-142	OD1 D30 B	2.20	16.36
7	N D29 A	H D29 A	O7 GRL-142	2.29	3.52
8	N D30 B	H D30 B	N1 GRL-142	2.38	1.86
9	N23 GRL-142	H39 GRL-142	OD2 D25 A	2.02	1.72
10	N23 GRL-142	H39 GRL-142	OD1 D25 A	2.00	1.24
11	N G48 B	H G48 B	N1 GRL-142	2.31	0.86
12	O26 GRL-142	H40 GRL-142	OD1 D25 B	2.33	0.24
13	N G48 B	H G48 B	N45 GRL-142	2.41	0.14
14	N I50 B	H I50 B	O34 GRL-142	2.08	0.04
15	NH1 R8 A	1HH1 R8 A	N1 GRL-142	2.67	0.02
16	N G49 B	H G49 B	O34 GRL-142	2.62	0.02
17	O26 GRL-142	H40 GRL-142	OD2 D25 B	2.55	0.02

Table S4: Hydrogen bonds Donor (D)–Hydrogen (H)$\cdots$Acceptor (A) between G and Mut in **Rep1** calculated using distance D-A$\leq$ 3.5 Å and angle D-H-A $\geq$ 150°. HB distance: average distance between Hydrogen and H-Acceptor. Occupancy: average occupancy calculated using 5000 frames from the trajectory. All distances in (Å). G labels in Figure 1.

Number	Donor	Hydrogen	Acceptor	HB distance	Occupancy (%)
1	N D29 A	H D29 A	O5 GRL-142	2.11	88.48
2	N D30 A	H D30 A	O7 GRL-142	2.18	81.46
3	O26 GRL-142	H40 GRL-142	OD2 D25 A	1.84	63.25
4	N I50 A	H I50 A	O35 GRL-142	2.21	35.93
5	O26 GRL-142	H40 GRL-142	OD1 D25 A	1.84	28.99
6	N I50 B	H I50 B	O34 GRL-142	2.15	18.98
7	N D29 A	H D29 A	O35 GRL-142	2.28	9.62
8	O26 GRL-142	H40 GRL-142	OD2 D25 B	2.10	5.16
9	N I50 A	H I50 A	O34 GRL-142	2.19	4.48
10	N23 GRL-142	H39 GRL-142	O G27 A	2.20	2.50
11	N45 GRL-142	H38 GRL-142	O P79 A	2.14	2.40
12	N I50 A	H I50 A	O5 GRL-142	2.26	0.58
13	N45 GRL-142	H38 GRL-142	O D30 B	2.44	0.38
14	N G48 B	H G48 B	N1 GRL-142	2.29	0.34
15	N I50 B	H I50 B	O35 GRL-142	2.29	0.32
16	N23 GRL-142	H39 GRL-142	N A28 A	2.51	0.26
17	NH1 R8 A	1HH1 R8 A	N1 GRL-142	2.08	0.12
18	N23 GRL-142	H39 GRL-142	OD2 D25 A	2.36	0.12
19	N G48 B	H G48 B	N45 GRL-142	2.47	0.08
20	NH2 R8 B	1HH2 R8 B	O7 GRL-142	2.17	0.06
21	N A28 A	H A28 A	O26 GRL-142	2.54	0.04
22	N D30 A	H D30 A	O5 GRL-142	2.45	0.04
23	OG1 T80 A	HG1 T80 A	N1 GRL-142	1.85	0.02
24	NH2 R8 B	1HH2 R8 B	O5 GRL-142	1.94	0.02
25	N D29 B	H D29 B	N45 GRL-142	2.51	0.02
26	N D30 B	H D30 B	N1 GRL-142	2.51	0.02
27	N G49 B	H G49 B	O34 GRL-142	1.90	0.02
28	N I50 B	H I50 B	O5 GRL-142	2.46	0.02
29	N45 GRL-142	H38 GRL-142	OD2 D30 B	2.40	0.02
30	N45 GRL-142	H38 GRL-142	O L46 B	2.49	0.02
31	N45 GRL-142	H38 GRL-142	O G48 B	2.04	0.02
32	N I50 A	H I50 A	O5 GRL-142	2.40	0.02

Table S5: Hydrogen bonds Donor (D)–Hydrogen (H)···Acceptor (A) between G and Mut in **Rep2** calculated using distance D-A$\leq$ 3.5 Å and angle D-H-A $\geq$ 150°. HB distance: average distance between Hydrogen and H-Acceptor. Occupancy: average occupancy calculated using 5000 frames from the trajectory. All distances in (Å). G labels in Figure 1.

Number	Donor	Hydrogen	Acceptor	HB distance	Occupancy (%)
1	N D29 A	H D29 A	O5 GRL-142	2.16	77.96
2	N D30 A	H D30 A	O7 GRL-142	2.24	70.19
3	N I50 B	H I50 B	O34 GRL-142	2.07	37.59
4	N I50 A	H I50 A	O5 GRL-142	2.30	37.01
5	O26 GRL-142	H40 GRL-142	OD2 D25 A	1.86	33.81
6	N D30 B	H D30 B	N1 GRL-142	2.37	18.38
7	O26 GRL-142	H40 GRL-142	OD1 D25 B	2.13	12.98
8	N D29 A	H D29 A	O7 GRL-142	2.29	8.56
9	N45 GRL-142	H38 GRL-142	OD1 D30 B	2.27	7.32
10	N45 GRL-142	H38 GRL-142	OD2 D30 B	2.28	5.52
11	N I50 B	H I50 B	O35 GRL-142	2.33	4.60
12	N45 GRL-142	H38 GRL-142	O P79 A	2.13	3.66
13	O26 GRL-142	H40 GRL-142	OD1 D25 A	1.89	2.84
14	N D29 B	H D29 B	N1 GRL-142	2.47	2.06
15	O26 GRL-142	H40 GRL-142	OD2 D25 B	2.27	1.04
16	N23 GRL-142	H39 GRL-142	OD2 D25 A	1.93	0.90
17	N I50 B	H I50 B	O5 GRL-142	2.16	0.58
18	NH1 R8 A	1HH1 R8 A	N1 GRL-142	2.23	0.54
19	NH2 R8 A	1HH2 R8 A	N1 GRL-142	2.24	0.48
20	N23 GRL-142	H39 GRL-142	OD1 D25 A	2.17	0.48
21	N45 GRL-142	H38 GRL-142	NH2 R8 A	2.46	0.32
22	N45 GRL-142	H38 GRL-142	NH1 R8 A	2.45	0.18
23	NH1 R8 B	1HH1 R8 B	O5 GRL-142	2.31	0.12
24	N23 GRL-142	H39 GRL-142	O G27 A	2.03	0.12
25	N G48 B	H G48 B	N1 GRL-142	2.41	0.08
26	N45 GRL-142	H38 GRL-142	OD1 D29 B	2.15	0.06
27	N45 GRL-142	H38 GRL-142	OD2 D29 B	2.14	0.06
28	N D30 A	H D30 A	O5 GRL-142	2.45	0.06
29	NH2 R8 B	1HH2 R8 B	O5 GRL-142	2.30	0.04
30	N I50 A	H I50 A	O34 GRL-142	2.37	0.04
31	N G48 B	H G48 B	N45 GRL-142	2.44	0.02
32	O26 GRL-142	H40 GRL-142	N A28 B	2.65	0.02

4.2.2 Water-mediated interactions

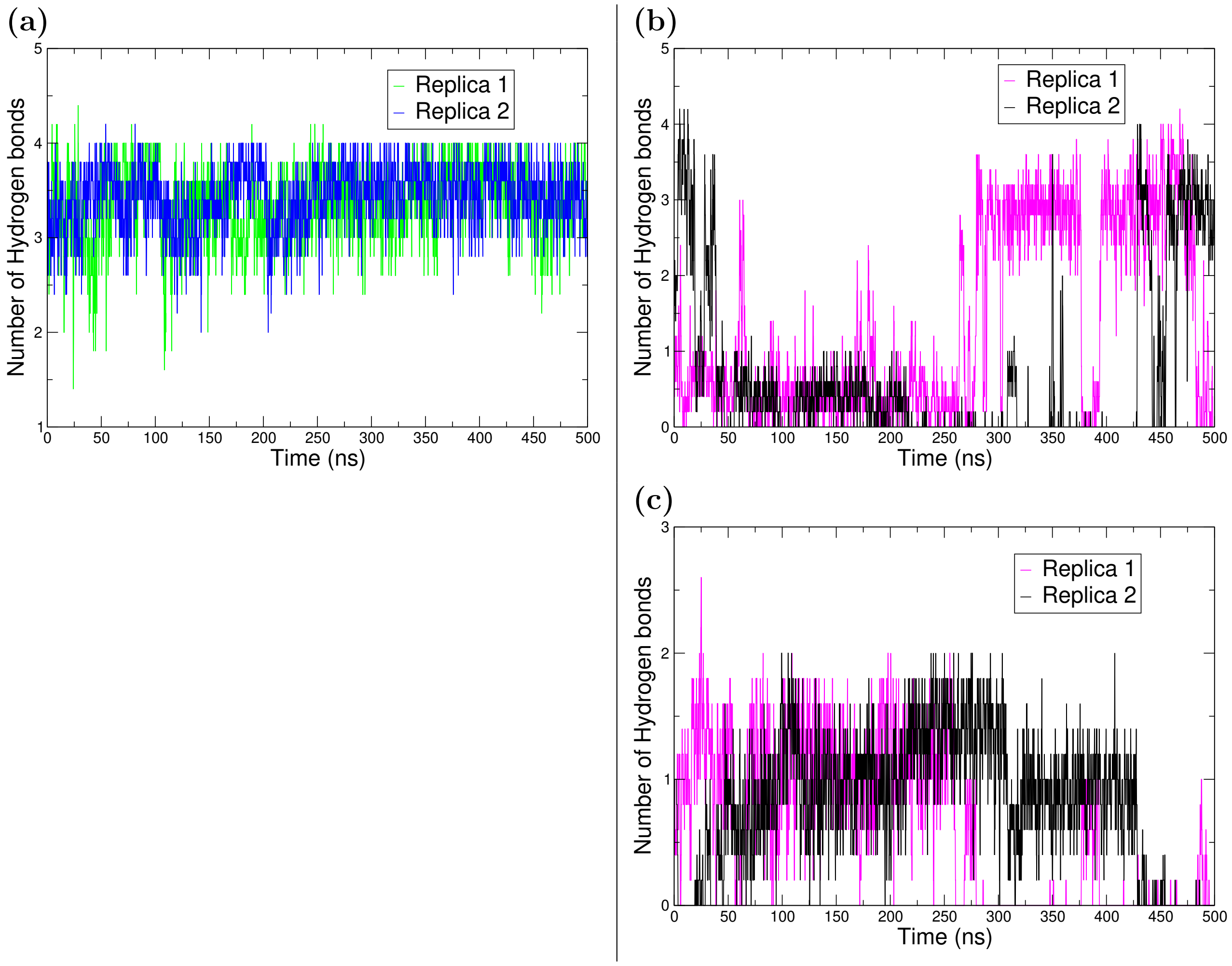

Figure S9: Monitoring of the 4 HBs mediated by the flap-water in Wt–G (a) and Mut–G (b). Average number of protease$\cdots$water$\cdots$ligand during the simulations. A marked disruption of the HB network can be observed in Mut–G. (c) Compensating HBs between the ligand and the main chain N-H groups of I50 and I50’.

4.2.3 Fluorine-mediated interactions

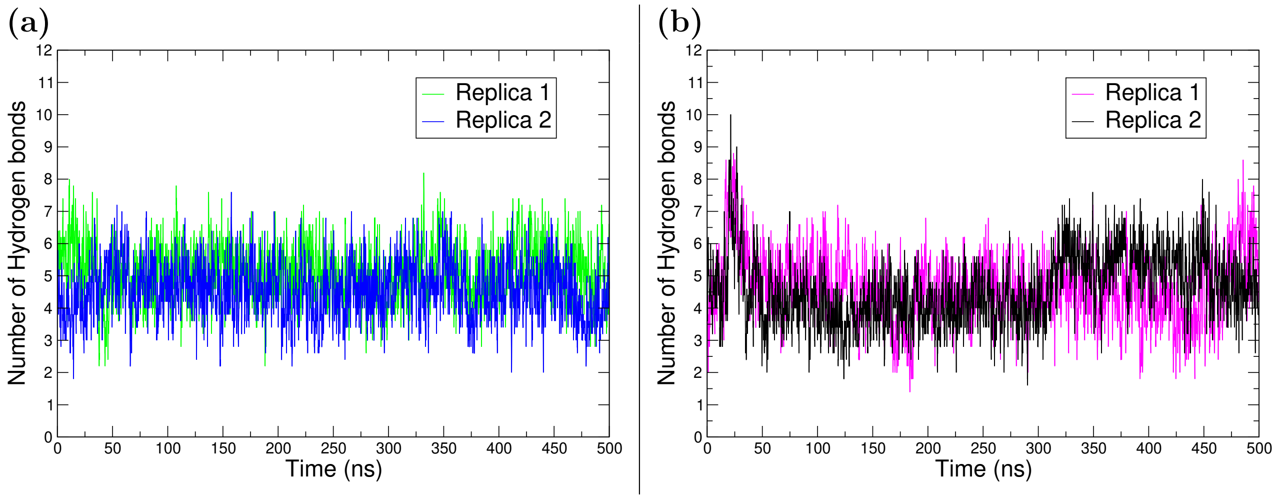

Figure S10: (a-b) Number of Donor (D)–Hydrogen (H)$\cdots$Fluorine (F) interactions, calculated using cutoffs for distance (D-F) $\leq$ 4.6 Å and angle (D-H-F) $\geq$ 120°. **Wt-G** Rep1: 5.1 ± 1.7 and Rep2: 4.6 ± 1.7; **Mut-G** Rep1: 4.6 ± 1.8 and Rep2: 4.6 ± 1.7.

4.2.4 Effect of specific mutations L33F and I54M

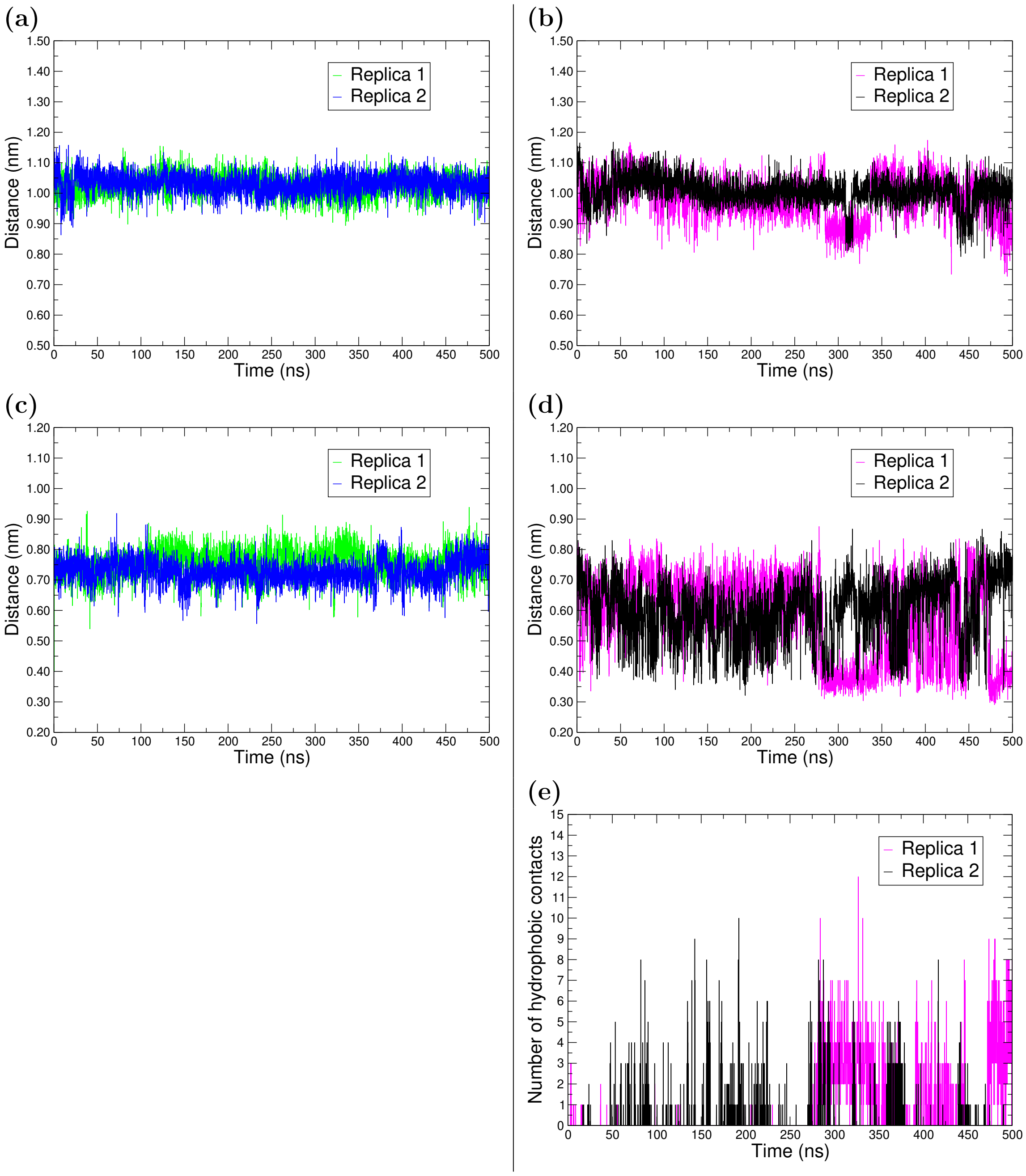

Figure S11: Monitoring of residues 33 and 54 in Wt–G and Mut–G. (a-b) Conserved hydrophobic interactions between residues 33 and G, in Wt–G and Mut–G. (c-d) Enhanced M54-side$\cdots$G hydrophobic interactions in Mut–G compared to the corresponding Wt–G. (e) Emerging M54-side$\cdots$G hydrophobic contacts that are absent in Wt–G.

5 MMPBSA binding energies

5.1 Total energies by region, group and binding mode

Table S6: Data used for the weighted-average BE calculation of Mut–G Rep1. "Contribution" = BE × occ/group.

Region	Init (ns)	Final (ns)	Duration (ns)	Occ/traj	Occ/traj (%)	BE kcal mol^{-1}	Contribution
R1	16	33	17	0.034	3.4	-23.50	-1.78
R2	34	49	15	0.030	3.0	-14.00	-0.94
R3	67	259	192	0.384	38.4	-16.90	-14.50
R4	285	336	51	0.102	10.2	-16.30	-16.30
R5	400	470	70	0.140	14.0	-10.20	-10.20
R6	470	500	30	0.060	6.0	-12.00	-12.00
						Sum of weights (sum occ/group)	4.00
						Sum of contributions	-55.71
Weighted average (replica-level) BE = (sum contributions) / (sum weights)							**-13.93 kcal mol^{-1}**

Group	Occurrences	Representative BE (kcal mol^{-1})	occ/mode (relative)
Group 1	224	-17.21	1.00
Group 2	51	-16.30	0.34
Group 3	70	-10.20	0.46
Group 4	30	-12.00	0.20
Total occurrences	375		
Weighted average (group-level) BE = $\sum$(occ × BE) / total occ			**-15.36 kcal mol^{-1}**
Native-like mode (224 occ)		-17.21	
Flap mode (151 occ)		-12.62	
Mode-level weighted average (224 vs 151)			**-15.36 kcal mol^{-1}**

Table S7: Data used for the weighted-average BE calculation of Mut–G Rep2. "Contribution" = BE × occ/group.

Region	Init (ns)	Final (ns)	Duration (ns)	Occ/traj	Occ/traj (%)	BE kcal mol^{-1}	Contribution
R1	46	55	9	0.018	1.8	-16.3	-0.76
R2	99	112	13	0.026	2.6	-17.5	-3.99
R3	217	300	83	0.166	16.6	-22.9	-9.90
R4	55	99	44	0.088	8.8	-21.3	-16.44
R5	116	216	100	0.200	20.0	-25.3	-13.17
R6	324	433	109	0.218	21.8	-24.6	-24.60
R7	438	500	62	0.124	12.4	-12.9	-12.90
						Sum of weights (sum occ/group)	4.00
						Sum of contributions	-81.76
Weighted average (replica-level) BE = (sum contributions) / (sum weights)						**-20.44 kcal mol^{-1}**	

Group	Occurrences	Representative BE (kcal mol^{-1})	occ/mode (relative)
Group 1	192	-23.84	0.76
Group 2	57	-20.43	0.34
Group 3	109	-24.60	0.66
Group 4	62	-12.90	0.24
Total occurrences	420		
Weighted average (group-level) BE = $\sum$(occ × BE) / total occ			**-21.93 kcal mol^{-1}**
Native-like mode (166 occ)		-23.17	
Flap mode (254 occ)		-21.17	
Mode-level weighted average (166 vs 254)			**-21.93 kcal mol^{-1}**

6 Analysis of the results of Replica 3 for Mut–G

Table S8: Binding free energies for Rep 3 of Mut–G computed with MMPBSA. All energies are reported in kcal/mol. Entropy corrections were carried out via normal mode analysis.

Energy contribution[a]	Mut–G Rep3
ΔE_{vdw}	$-69.00(4.53)^b(0.23)^c$
ΔE_{ele}	$-19.36(6.86)(0.34)$
ΔG_{pol}	$46.15(6.47)(0.32)$
ΔG_{nonpol}	$-6.47(0.23)(0.01)$
ΔH	$-42.19(6.32)(0.32)$
$-T\Delta S$	$30.87(6.02)(0.30)$
ΔG	$-11.32(8.78)(0.44)$

[a] Binding energies were computed throughout the entire 1 μs trajectory.
[b] Standard deviation (SD).
[c] Standard error of the mean (SEM).

Although the ligand RMSD of Rep3 exhibits noticible fluctuations (See Figure S1 (b)), no native-like conformations are observed. Instead, the ligand P2' group rapidly abandons its

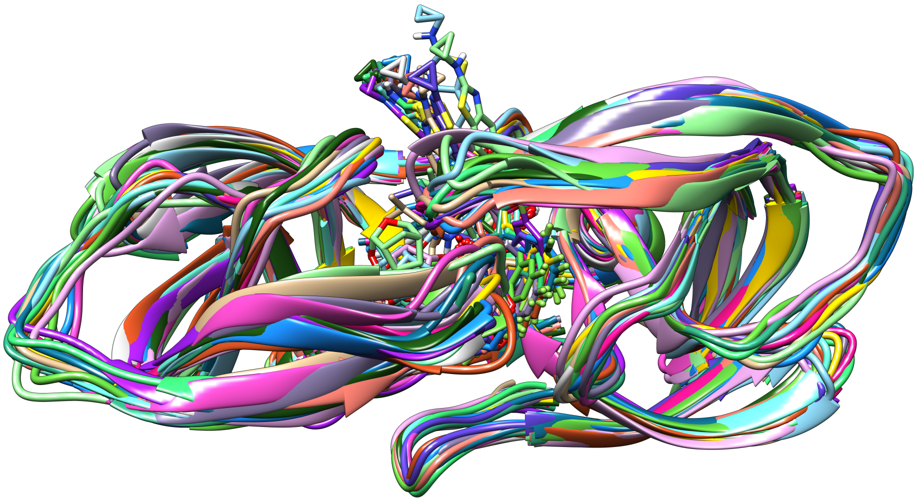

Figure S12: Superposition of Mut–G snapshots (Top view) taken each 50 ns during the entire 1 μs trajectory, showing permanent detachment of P2' moiety.

subsite and points outward with respect to the active site. This behavior is clearly illustrated in Figure S12, which shows a superposition of snapshots taken each 50 ns over the entire 1 μs trajectory. The absence of conformations close to the native binding pose may explain the relatively low binding energy compared to replicas 1 and 2, in which a variety of native-like conformations are attained.

7 MD simulations of GRL-142 in pure water

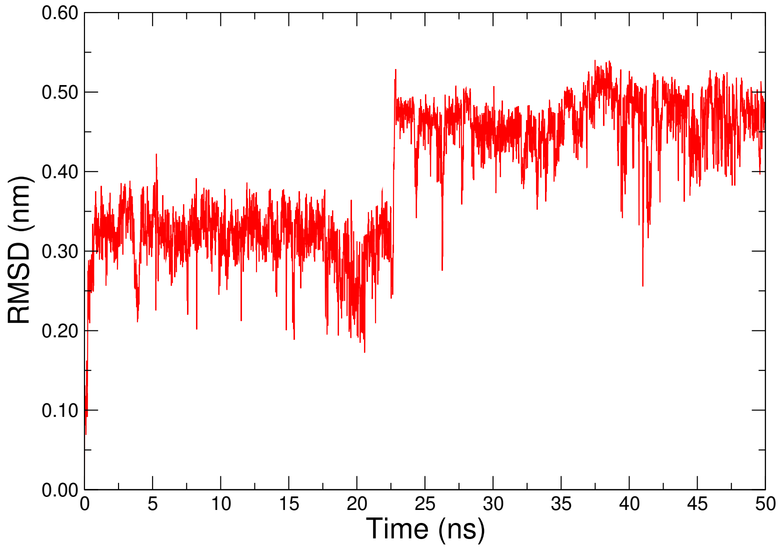

Figure S13: RMSD of G during 50 ns production in pure water.

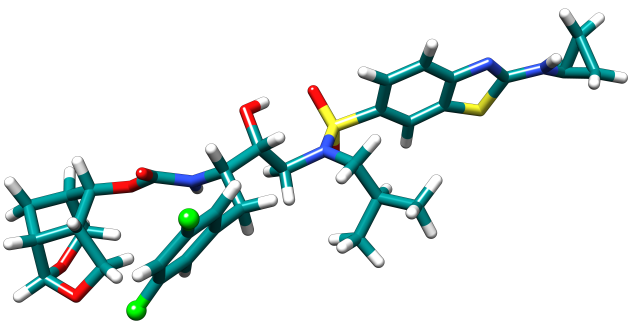

(a) 3 ns

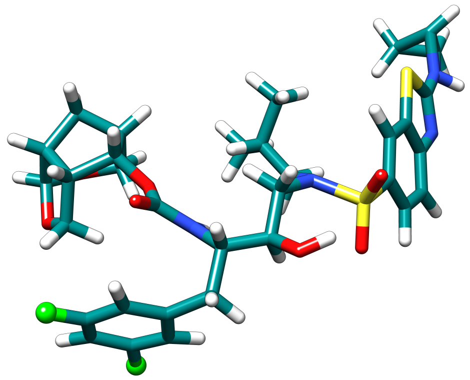

(b) 9 ns

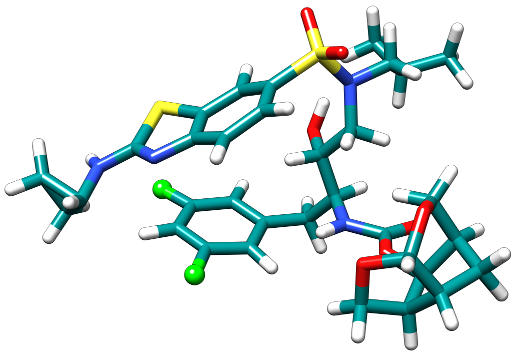

(c) 26 ns

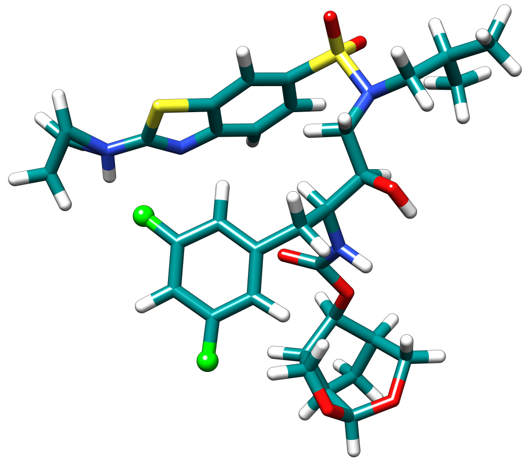

(d) 40 ns

Figure S14: Conformations of G in pure water along the 50 ns trajectory.

References

[1] Grosdidier, A.; Zoete, V.; Michielin, O. SwissDock, a protein-small molecule docking web service based on EADock DSS. *Nucleic acids research* **2011**, *39*, W270–W277.

[2] Rohrig, U. F.; Goullieux, M.; Bugnon, M.; Zoete, V. Attracting cavities 2.0: improving the flexibility and robustness for small-molecule docking. *Journal of chemical information and modeling* **2023**, *63*, 3925–3940.

[3] Pettersen, E. F.; Goddard, T. D.; Huang, C. C.; Couch, G. S.; Greenblatt, D. M.; Meng, E. C.; Ferrin, T. E. UCSF Chimera—a visualization system for exploratory research and analysis. *J. Comput. Chem.* **2004**, *25*, 1605–1612.

[4] Eberhardt, J.; Santos-Martins, D.; Tillack, A. F.; Forli, S. AutoDock Vina 1.2. 0: new docking methods, expanded force field, and python bindings. *Journal of chemical information and modeling* **2021**, *61*, 3891–3898.

[5] Alonso, H.; Bliznyuk, A. A.; Gready, J. E. Combining docking and molecular dynamic simulations in drug design. *Medicinal research reviews* **2006**, *26*, 531–568.

[6] Salmaso, V.; Moro, S. Bridging molecular docking to molecular dynamics in exploring ligand-protein recognition process: An overview. *Front. Pharmacol.* **2018**, *9*, 923.

[7] Fan, J.; Fu, A.; Zhang, L. Progress in molecular docking. *Quant. Biol.* **2019**, *7*, 83–89.

[8] Ghosh, A. K.; Martyr, C. D.; Osswald, H. L.; Sheri, V. R.; Kassekert, L. A.; Chen, S.; Agniswamy, J.; Wang, Y.-F.; Hayashi, H.; Aoki, M.; others Design of HIV-1 Protease Inhibitors with Amino-bis-tetrahydrofuran Derivatives as P2-Ligands to Enhance Backbone-Binding Interactions: Synthesis, Biological Evaluation, and Protein–Ligand X-ray Studies. *Journal of medicinal chemistry* **2015**, *58*, 6994–7006.

[9] Ghosh, A. K.; Weber, I. T.; Mitsuya, H. Beyond darunavir: recent development of next generation HIV-1 protease inhibitors to combat drug resistance. *Chemical Communications* **2022**, *58*, 11762–11782.
